# Functional Traits and Spatio-Temporal Structure of a Major Group of Soil Protists (Rhizaria: Cercozoa) in a Temperate Grassland

**DOI:** 10.3389/fmicb.2019.01332

**Published:** 2019-06-11

**Authors:** Anna Maria Fiore-Donno, Tim Richter-Heitmann, Florine Degrune, Kenneth Dumack, Kathleen M. Regan, Sven Marhan, Runa S. Boeddinghaus, Matthias C. Rillig, Michael W. Friedrich, Ellen Kandeler, Michael Bonkowski

**Affiliations:** ^1^Terrestrial Ecology Group, Institute of Zoology, University of Cologne, Cologne, Germany; ^2^Cluster of Excellence on Plant Sciences (CEPLAS), Cologne, Germany; ^3^Microbial Ecophysiology Group, Faculty of Biology/Chemistry, University of Bremen, Bremen, Germany; ^4^Institute of Biology, Plant Ecology, Freie Universität Berlin, Berlin, Germany; ^5^Berlin-Brandenburg Institute of Advanced Biodiversity Research, Berlin, Germany; ^6^The Ecosystems Center, Marine Biological Laboratory, Woods Hole, MA, United States; ^7^Department of Soil Biology, Institute of Soil Science and Land Evaluation, University of Hohenheim, Stuttgart, Germany

**Keywords:** biogeography, functional traits, soil ecology, protozoa, microbial assembly, environmental selection, dispersal limitation, soil protists

## Abstract

Soil protists are increasingly appreciated as essential components of soil foodwebs; however, there is a dearth of information on the factors structuring their communities. Here we investigate the importance of different biotic and abiotic factors as key drivers of spatial and seasonal distribution of protistan communities. We conducted an intensive survey of a 10 m^2^ grassland plot in Germany, focusing on a major group of protists, the Cercozoa. From 177 soil samples, collected from April to November, we obtained 694 Operational Taxonomy Units representing >6 million Illumina reads. All major cercozoan taxonomic and functional groups were present, dominated by the small flagellates of the Glissomonadida. We found evidence of environmental selection structuring the cercozoan communities both spatially and seasonally. Spatial analyses indicated that communities were correlated within a range of 3.5 m. Seasonal variations in the abundance of bacterivores and bacteria, followed by that of omnivores suggested a dynamic prey-predator succession. The most influential edaphic properties were moisture and clay content, which differentially affected each functional group. Our study is based on an intense sampling of protists at a small scale, thus providing a detailed description of the biodiversity of different taxa/functional groups and the ecological processes involved in shaping their distribution.

## Introduction

Our understanding of soil ecosystem functioning relies on a clear image of the drivers of the diverse interactions occurring among plants and the components of the soil microbiome – bacteria, fungi, and protists. Protists are increasingly appreciated as important components of soil foodwebs (Bonkowski et al., 2019). Their varied and taxon-specific feeding habits differentially shape the communities of bacteria, fungi, algae, small animals, and other protists (Geisen, 2016; Trap et al., 2016). However, soil protistology is presently less advanced than its bacterial or fungal counterpart, and there is a dearth of information on the factors structuring protistan communities: this may be due to the polyphyly of protists, an immensely heterogeneous assemblage of distantly related unicellular organisms, featuring a vast array of functional traits (Pawlowski et al., 2012). Trait-based approaches offer opportunities to gain a deeper understanding of the interactions between microbial diversity and ecosystem functioning (Krause et al., 2014), but have rarely been applied to protists.

Assessing how microbial diversity contributes to ecosystem functioning requires the identification of appropriate spatial scales at which biogeographies can be predicted and at which the roles of homogenizing or selective biotic and abiotic processes can be characterized. Local contemporary habitat conditions appear to be the most important deterministic factors shaping bacterial biogeographies, although assembly mechanisms might differ between different taxonomic or functional groups (Lindström and Langenheder, 2012; Karimi et al., 2018). Spatial distribution of microorganisms is driven by different factors at different scales (Meyer et al., 2018). At a macroecological scale, soil microbial communities are shaped mainly by abiotic factors. Bacteria and archaea, in particular, are mostly influenced by pH (Kaiser et al., 2016; Meyer et al., 2018), while the diversity and composition of fungal communities seem to be driven by moisture and nutrient availability (Peay et al., 2016). At a smaller scale, soil moisture influences bacteria (Brockett et al., 2012; Serna-Chavez et al., 2013), while historical contingency and competitive interactions seem to shape fungal communities (Peay et al., 2016). Seasonal variability is a major factor driving prokaryotic communities (Lauber et al., 2013), and this has been confirmed at our study site for both bacteria and archaea (Regan et al., 2014, 2017; Stempfhuber et al., 2016).

However, due to high microbial turnover rates as well as their capacity for passive dispersal, a large proportion of variation in microbial community assembly can be ascribed to stochastic processes rather than deterministic ones (Nemergut et al., 2013), although to what degree is unclear (Dini-Andreote et al., 2015). However, it is well known that soil abiotic and biotic factors contribute to deterministic community assembly (Zhou and Ning, 2017). Deterministic patterns of community assembly can result from species sorting along a pH gradient (Karimi et al., 2018), habitat filtering by root exudates resulting in selection of phylogenetically clustered microbiomes (Sapp et al., 2018; Perez-Jaramillo et al., 2016), or patch dynamics due to trade-offs between r- and K-selected communities driven by differences in soil organic matter quality (Christensen et al., 1992; Fierer et al., 2007).

Although protistan communities have been biogeographically characterized at large scales (Bates et al., 2013), some authors suggested that their assembly could be entirely driven by stochastic processes (Bahram et al., 2016; Zinger et al., 2018). We hypothesized that by providing a thorough sampling of protistan communities in soil, we would demonstrate that spatial and temporal abiotic and biotic processes significantly contributed to protistan community assembly. In particular, among abiotic factors, soil moisture (Bates et al., 2013; Lentendu et al., 2014), pH, carbon, and nitrogen (Lentendu et al., 2014; Lanzén et al., 2016) have been shown to significantly influence protistan communities. We expected patterns of seasonality (Lara et al., 2011), perhaps shaped by biotic factors such as bacteria and vegetation, although with nuanced effects related to functional groups or lineages.

Our study site was located in a mountain range in southwest Germany and was part of a larger interdisciplinary project of the German Biodiversity Exploratories (Fischer et al., 2010). We applied a MiSeq Illumina sequencing protocol using barcoded primers amplifying a c. 350 bp fragment of the hypervariable region V4 of the small subunit ribosomal RNA gene (SSU or 18S) (Fiore-Donno et al., 2018). We focused on Cercozoa (Cavalier-Smith, 1998) as an example of a major protistan lineage in soil (Urich et al., 2008; Domonell et al., 2013; Turner et al., 2013; Geisen et al., 2015; Grossmann et al., 2016). This highly diverse phylum [c. 600 described species, (Pawlowski et al., 2012)] comprises a vast array of functional traits in morphologies, nutrition and locomotion modes, and thus can represent the diversity of soil protists. We provided a functional trait-based classification of cercozoan and related endomyxan taxa found in our survey. We explored in a small, unfertilized grassland plot, how spatial distance, season, and edaphic parameters (abiotic and biotic) shape the diversity and dynamics of the cercozoan communities.

## Materials and Methods

### Study Site, Soil Sampling, and DNA Extraction

The sampling site was located near the village of Wittlingen, Baden–Württemberg, in the Swabian Alb (“Schwäbische Alb”), a limestone middle mountain range in southwest Germany. Details of the sampling procedure are provided elsewhere (Regan et al., 2014; Stempfhuber et al., 2016). Briefly, a total of 360 samples were collected over a 6-month period from spring to late autumn in a 10 m^2^ grassland plot within the site AEG31 of the Biodiversity Exploratory Alb (48.42 N; 9.5 E), with a minimum distance of 0.45 m between two adjacent samples (Supplementary Figure S1). For this study, we selected 180 samples, 30 samples from each sampling date (April, May, June, August, October, and November 2011). We were provided the soil DNA, extracted from duplicate homogenized soil subsamples (300 mg each) as described in Regan et al. (2017). Soil physicochemical parameters were determined as described in Regan et al. (2014, 2015); the parameters included in our analyses are listed in Supplementary Table S1, with their seasonal variation illustrated in Supplementary Figures S2, S3. Over this area, spatial variability was limited; only the proportion of clay content varied (indicated in Supplementary Figure S2 by high boxes). Soil moisture changed most dramatically over the sampling period, with a peak in April and lowest values in May and October (average = 40%, max = 63%, min = 23%, SD = 11). Microbial biomass-related carbon and nitrogen parameters peaked in April. Bacterial cell counts showed a distinct peak in April. Living plant and fungi-related parameters followed the seasonal pattern of a minimum after winter, a maximum in summer, and a decrease in autumn (for the plant biomass, after mowing in August), with plant litter biomass following an inverse trend (Supplementary Figure S2).

### Amplification, Library Preparation, and Sequencing

Primer design, barcoding primers, amplification, library preparation, and Illumina sequencing have been described in detail (Fiore-Donno et al., 2018). The primers covered nearly the total diversity of Cercozoa, although they were biased against Endomyxa (mostly parasitic lineages). The primers amplified a fragment of 335–544 bp of the hypervariable region V4 of the SSU. Briefly, amplicons were obtained in two successive PCRs, the first using 1 μl of 1:10 soil DNA as template, the second using semi-nested primers and 1 μl of the first PCR as template. We employed the following final concentrations: GreenTaq polymerase (Fermentas, Canada) 0.01 units, buffer 1x, dNTPs 0.2 mM and primers 1 μM. The thermal program consisted of an initial denaturation step at 95°C for 2 min, 24 cycles at 95°C for 30 s, 50°C for 30 s, 72°C for 30 s; and a final elongation step at 72°C for 5 min. Barcoded primers were used in the second PCR to index samples. We pooled 15 μl of each of the successfully amplified samples (including the mock community, see below), then reduced the total volume to 80 μl; c. 800 ng of amplicons were used for the single library preparation as previously described (Fiore-Donno et al., 2018). The library concentration was 23 nM, of which 10 pM were used for the Illumina sequencing run. Sequencing was performed with a MiSeq v2 Reagent kit of 500 cycles on a MiSeq Desktop Sequencer (Illumina Inc., San Diego, CA, United States) at the University of Geneva (Switzerland), Department of Genetics and Evolution. To validate the bioinformatics pipeline (see below), we amplified DNA from a “mock community”, consisting of known representative Cercozoa (Fiore-Donno et al., 2018), plus *Cercomonas longicauda* provided by S. Flues (GenBank DQ442884), totaling 11 species.

### Sequence Processing

Paired reads were assembled following a published protocol (Lejzerowicz et al., 2014). The quality filtering discarded (i) all sequences with a mean Phred quality score < 30, shorter < 25 bases, with 1 or more ambiguities in the tag or in the sequence and with more than one ambiguity in the primers, and (ii) assembled sequences with a contig of < 100 bp and more than 10 mismatches (Table 1). Sequences were sorted by samples (“demultiplexing”) via detection of the barcodes (Supplementary Table S2; Fiore-Donno et al., 2018). The bioinformatics pipeline was optimized using the mock community. We first separated the sequences obtained from the 11 known taxa and ran the analysis with the steps listed in Table 1. The settings that made it possible to retrieve the expected 11 operational taxonomic units (hereafter OTUs) from the mock community were then applied to the environmental sequences.

**Table 1:** Number of reads retrieved at each step of the bioinformatic pipeline.

Mock community (11 taxa)	Unique	Genuine Cercozoa	Aligned	Non-chimeric	Clustered 97% sim.	OTUs rel. abundance ≥ 0.01
Representative sequences	8430	7582	7552	7319	182	11
All sequences	22818	14723	14693	14321	14321	14031
% removed	0	10	0.2	3	0	2

**Environmental reads**	**Trimmed**	**Unique**	**Clustered 97% sim. rel. abundance ≥ 0.01**	**Genuine, aligned, non-chimeric**	

Representative sequences	10052231	5556619	1324		694	
All sequences	10052231	10029413	7856763		6225241	
% removed		0	22		21	

*Initial number of paired reads = 15445807.*

Sequences were clustered into OTUs using vsearch v.1 (Rognes et al., 2016), with the abundance-based greedy clustering algorithm (agc) implemented in mothur v.3.9 (Schloss et al., 2009) with a similarity threshold of 97%. Using BLAST+ (Camacho et al., 2008) with an *e*-value of 1^-50^ and keeping only the best hit, OTUs were identified using the PR2 database (Guillou et al., 2013); non-cercozoan OTUs were removed. Chimeras were identified using UCHIME (Edgar et al., 2011) as implemented in mothur v.3.9 as previously described (Fiore-Donno et al., 2018); chimeras and misaligned sequences were removed (Table 1).

### Cercozoan Functional Traits

We selected three categories of ecological relevance: feeding mode, morphology, and locomotion mode. For the feeding mode, we classified the cercozoan and endomyxan OTUs into bacterivores, eukaryvores (feeding on algae, fungi, other protists, and small animals but with no reports of feeding on bacteria) and omnivores, feeding on both bacteria and eukaryotes, according to available information in the literature. We applied two criteria for morphological classification: (i) the presence or absence of a shell (testate or naked); (ii) if the cell was an amoeba, a flagellate or an amoeboflagellate. We retained existing combinations, consisting of five categories (Supplementary Table S3). We further distinguished two types of tests, organic or agglutinated – from those made of silica. The major difference in locomotion mode was set between cells creeping/gliding on substrate (on soil particles or on roots) versus free-swimming ones (in interstices filled with water). The phytomyxean parasites (Endomyxa), due to their peculiar life cycle, were considered separately in each functional category. We assigned traits at different taxonomic levels, from order to genus (Supplementary Table S3), and provide the respective references (Supplementary Table S4).

### Statistical and Phylogenetic Analyses

All statistical analyses were carried out within the R environment (R v. 3.5.1) (R Development Core Team, 2014). Community analyses were performed with the packages vegan (Oksanen et al., 2013) and betapart (Baselga, 2017). We used: (i) Mantel tests and correlograms for spatial analysis; (ii) redundancy analysis (RDA) and generalized linear mixed models (GLMM) to describe the influence of environmental factors on community and population levels; (iii) analysis of similarity (anosim), multi-permutation procedure (MRPP) and permutational multivariate analysis of variance (PERMANOVA) to analyze temporal variation at the community level; (iv) *post hoc* analysis (estimated marginal means on generalized least squares (GLS) with correction for spatial autocorrelation) to test temporal variation among taxonomic and functional groups; (v) variance partitioning to disentangle community turnover into spatial, temporal, and environmental components. For RDA and variance partitioning, stepwise forward selection based on the Akaike Information Criterion was used to identify important variables. For GLMMs, the same procedure was extended with subsequent averaging of candidate models to obtain highly sparse models. Cercozoan diversity was illustrated using the Sankey diagram generator V1.2^1^. Analyses are described in detail in Supplementary Data S1.

## Results

### Sequencing Results

We obtained over 15 million filtered, paired reads (Table 1). The rate of mistagging during the sequencing run (indicating cross-over between adjacent clusters) was low, only 0.92%. To retrieve the 11 OTUs from the mock community, OTUs represented by ≤ 0.01% of the total sequences had to be removed; thus, this cutoff was applied to the environmental sequences. Not applying this cutoff would have drastically inflated the number of OTUs retrieved from the mock community (182 OTUs instead of 11). The percentage of non-cercozoan/endomyxan OTUs accounted for only 7.63% of the total sequences, confirming the high specificity of the primers. We obtained 694 genuine cercozoan/endomyxan OTUs from 177 grassland soil samples representing 6225241 sequences (Table 1). Three samples could not be amplified (Supplementary Figure S1). An NMDS analysis indicated that an additional sample was an outlier, thus it was omitted from subsequent analyses. The average number of OTUs per sample was 637 (maximum 681, minimum 407, SE 38.2). The average of sequences/soil sample was 35171 (maximum 153794, minimum 9808, SE 18.415), leading to an estimation of the coexistence of an average of c. 1000 cercozoan OTUs per gram of soil. We provide a database with the OTU abundance in each sample, their taxonomic assignment and their estimated functional traits (Supplementary Tables S3, S4).

### Diversity of Cercozoa

**FIGURE 1:**
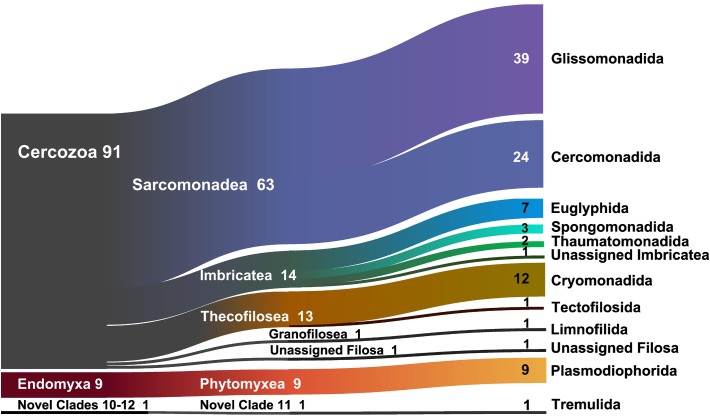
Sankey diagram showing the relative contribution of the OTUs to the taxonomic diversity. Taxonomical assignment is based on the best hit by BLAST. From left to right, names refer to phyla (Cercozoa, Endomyxa), class (ending -ea), and orders (ending -ida). “Unassigned” refer to sequences that could not be assigned to the next lower-ranking taxon or to “incertae sedis” order or families. Numbers are percentages of sequences’ abundances – taxa represented by <1% are not shown.

At a high taxonomic level, the majority of the 694 OTUs could be assigned to the phylum Cercozoa (91% of the sequences) (Figure 1), the remaining to Endomyxa (9%) and to the *incertae sedis* Novel clade 10 (Tremulida, 1%). Only 39% of the OTUs had 97–100% similarity to any known sequence in the PR2 database (Supplementary Figure S4). The 12 most abundant OTUs (>10000 sequences) accounted for 45% of the total sequences, while many low-abundance OTUs (243 < 1000 sequences) contributed to only 3% of the total sequences. These 12 OTUs were attributed to five orders: Glissomonadida (mostly Sandonidae), Cryomonadida (mostly two different *Rhogostoma* lineages), Plasmodiophorida (mostly the parasitic *Polymyxa graminis*), Cercomonadida (*Eocercomonas* and *Paracercomonas*), and Spongomonadida. The phylogenetic tree (Supplementary Figure S5) obtained with 176 reference sequences and 694 OTUs was rooted with Phytomyxea (Endomyxa); the vampyrellids (Proteomyxidea) and the Novel Clades 10-11-12, were paraphyletic to the monophyletic Cercozoa (93%). In Cercozoa, the main clades were recovered, although with low support. We were able to recover environmental sequences from nearly every clade of the tree.

The rarefaction curve including all samples reached a plateau, suggesting that the global richness of 694 OTUs could have been obtained by c. 70000 sequences (Supplementary Figure S6A), and by only 15 samples (Supplementary Figure S6B), and that the observed distribution patterns would not have been influenced by undersampling. Most OTUs were present in all sites (only 8.15% of absences in Supplementary Table S2). Multiple site beta diversity, calculated with either presence-absence or abundance on both rarefied and relative data, showed minor variation between the six sampling dates, which was confirmed by a resampling approach of random subsets (average resampled Bray–Curtis distances comprised between 0.715 and 0.745) (Supplementary Table S5).

### Functional Diversity

**FIGURE 2:**
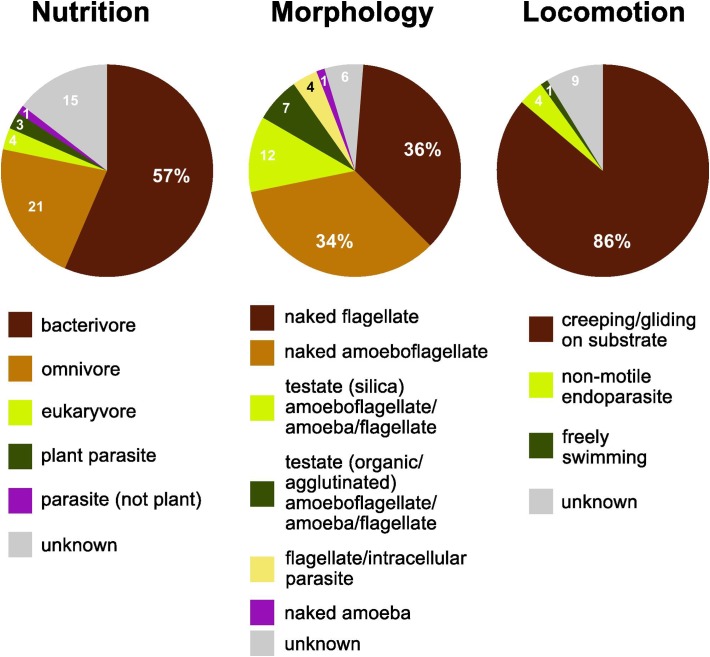
Functional diversity of cercozoan taxa. The relative proportions of functional groups classified according to nutrition, morphology, and locomotion modes.

More than half of the soil cercozoan OTUs were considered to be bacterivores (57%), followed by omnivores (21%), and eukaryvores (4%). Plant parasites (3%) and parasites of Oomycota (1%) were only marginally represented (Figure 2). Naked flagellates (36%) or amoeboflagellates (34%) together constituted the majority of the morphotypes, whereas testate cells (organic/agglutinated or siliceous) were less frequent, 12 and 7%, respectively. Naked amoebae were only marginally represented (1%). The dominant locomotion mode was creeping/gliding on substrate (86%), with only 1% free-swimming.

### Spatial Structuring and Seasonal Variation

**FIGURE 3:**
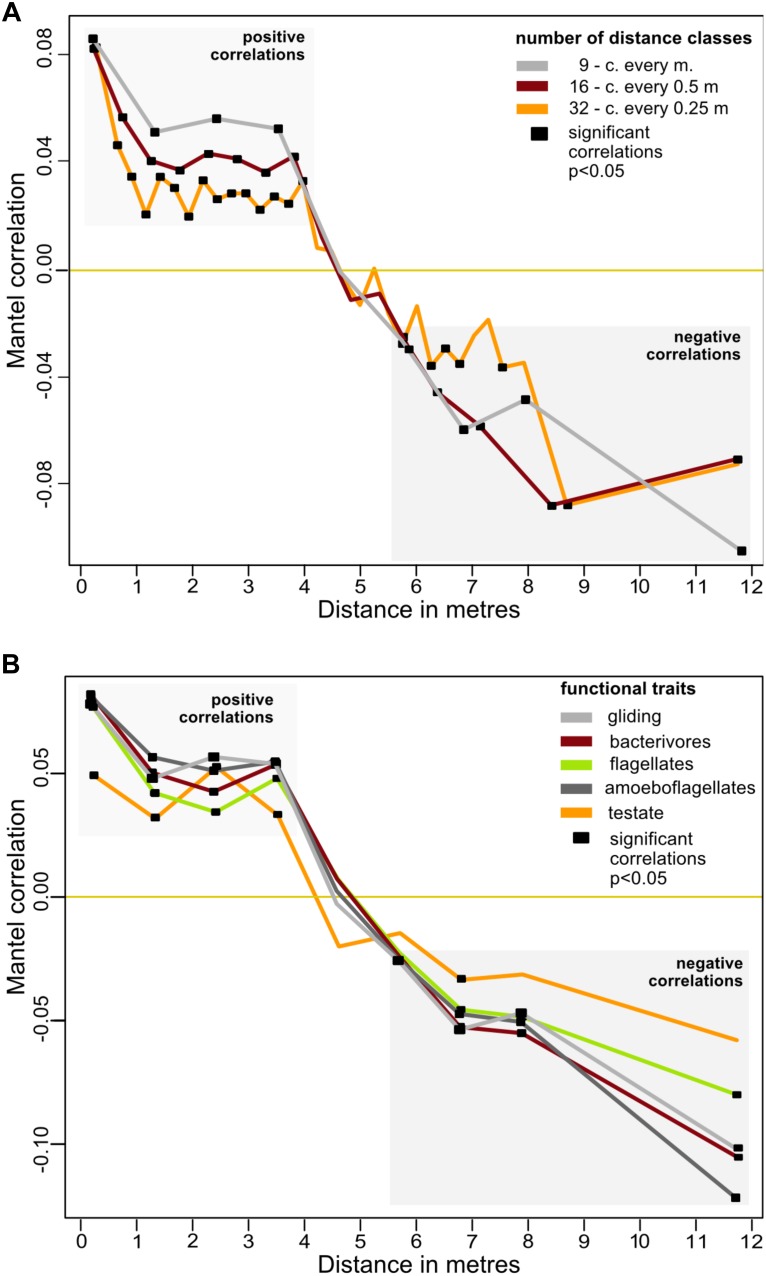
Effect of distance on the beta-diversity of the cercozoan communities. **(A)** Mantel correlograms based on Bray-Curtis OTUs dissimilarities compared to Euclidian spatial distances and the Spearman correlation coefficient. Three distance classes were considered, resulting in intervals of 0.25, 0.5, and 1 m. Only significant values (*p* < 0.05) are highlighted with black squares. Positive correlations were detected at distances from 0.45 to 3.875 m, no correlations at distances between 4.0 and 5.5 m. From 5.5 to 12.4 m, the communities were negatively correlated. **(B)** Mantel correlograms as above, calculated for OTUs grouped by the most represented functional traits, with nine distance classes.

The spatial distribution of the cercozoan communities was non-random for all OTUs (Mantel *R* = 0.1661, *p* = 0.0001), and also for all functional groups considered (Table 2). Mantel correlograms indicated that similarities among communities decreased with distance, although the coefficients were low (Mantel correlograms, Figure 3 and Table 2). At distances between 0.45 to 3.9 m, cercozoan communities showed positive autocorrelations, whereas communities at distances between 5.5 and 12 m were more dissimilar (i.e., negatively correlated). No spatial autocorrelations were observed at distances ranging from 4 to 5.5 m (Figure 3). These results were reproducible at different distance classes, i.e., at intervals of 0.25, 0.5, and 1 m (Figure 3A). Similar results were obtained when functional traits were considered (Figure 3B).

**Table 2:** Spatial correlations and effect of distance on beta-diversity.

A	Mantel test		B	Mantel correlograms			
	Mantel R	*p* value (corrected)		Smallest distance	*p* val. corr.	Largest distance	*p* val. corr.
All OTUs	0.1661	0.0001	All OTUs, 9 classes	0.45	0.0001	3.5	0.0008
			All OTUs, 16 classes	0.45	0.0001	3.75	0.0024
			All OTUs, 32 classes	0.45	0.0001	3.875	0.0198
**Functional traits:**			**Functional traits:**				
Gliding on substrate	0.1609	0.0001	Gliding on substrate	0.45	0.0001	3.5	0.0008
Bacterivores	0.1642	0.0001	Bacterivores	0.45	0.0001	3.5	0.001
Flagellates	0.1384	0.0001	Flagellates	0.45	0.0001	3.5	0.0032
Amoeboflagellates	0.1714	0.0001	Amoeboflagellates	0.45	0.0001	3.5	0.0008
Testate	0.0941	0.0002	Testate	0.45	0.0001	3.5	0.0274
**By seasons:**			**By seasons:**				
April	0.2702	0.0022	April	0.45	0.001	3.5	0.0176
May	0.1141	0.1041	May	Mantel not significant			
June	0.2608	0.001	June	0.45	0.0001	–	–
August	0.1654	0.0183	August	0.45	0.0001	2.5	0.0136
October	0.1239	0.0332	October	0.45	0.0001	1.5	0.0103
November	0.1121	0.0791	November	0.45	0.0008	1.5	0.0277

*A: Mantel tests (9999 permutations, Spearman correlation coefficient) calculated between the Bray–Curtis dissimilarity matrices and the Euclidian distances between sample coordinates. B: Mantel correlograms (9999 permutations, Spearman correlation coefficient) calculated with definite distance classes. Since results among 9, 16, and 32 classes were comparable for all OTUs, correlograms for OTUs grouped by functional traits and seasons were calculated using 9 classes. Positive correlations were found from the minimal distances between samples (0.45 m) to distances up to 3.875 m (Figure 3).*

**FIGURE 4:**
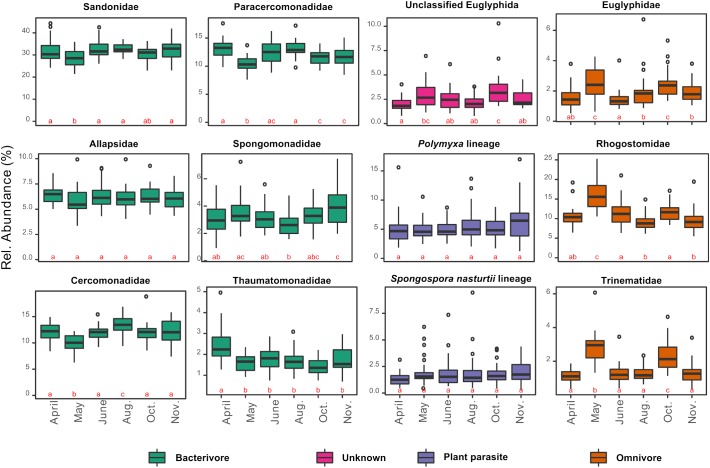
Box plots of the seasonal variation in the relative abundance of the 12 most abundant cercozoan families characterized by their nutrition mode (in different colors). Note that the *y*-scale varies between graphs. The small letters a, b, and c designate statistically significant differences in abundance between sampling dates (contrasts of estimated marginal means on generalized least squares models after correction for spatial autocorrelation). A change of letter from “a” to “b”, or “c” indicates a significant difference between sampling dates; “ab” indicate non-significant differences between dates sharing those letters.

In contrast to the overall homogeneity of beta diversity, cercozoan community structure changed significantly over time (anosim: *R* = 0.22, *p* < 0.001; PERMANOVA: *F*_5–170_ = 5.01, *p* < 0.001; MRPP: *p* < 0.001), although ordination (PCoA) suggested that the community turnover between April and May was most influential (Supplementary Figure S3). The cercozoan communities, binned by families or functional groups, showed distinct seasonal patterns of relative abundance (Figure 4). While the bacterivorous families peaked in April and decreased in May (except Spongomonadidae), the omnivorous, shell bearing Euglyphidae, Rhogostomidae, and Trinematidae exhibited an opposite pattern, increasing from April to May. The relative abundance of endomyxan plant parasites (*Polymyxa* and *Spongospora*) did not differ over the sampling season.

### The Main Driver of Cercozoan Species Turnover: Season, Distance, or Soil Parameters?

**FIGURE 5:**
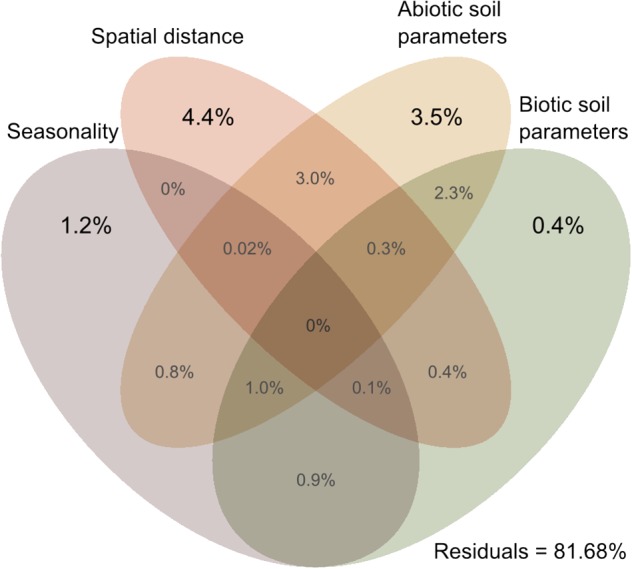
Venn diagram of variance partitioning analysis illustrating the effect of distance, season, and environment (biotic and abiotic edaphic factors) on the cercozoan communities. Values show the percentage of explained variance by each set of variables, and of joined effects in the intersections.

Cercozoan beta diversity was influenced by soil parameters (shown in Supplementary Table S1) (RDA: *F*_11–164_ = 3.3301, *p* = 0.001), spatial distance (RDA: *F*_6–169_ = 3.0202, *p* < 0.001), and seasonality (RDA: *F*_3–172_ = 3.389, *p* < 0.001). Variance partitioning among the four predictors indicated that biotic and abiotic soil parameters, spatial distance, and seasonality together accounted for 18.32% (adjusted *R*^2^) of the total variation in cercozoan beta diversity (Figure 5). Spatial distance explained 4.4% of the variation, followed by abiotic soil parameters (3.5%), seasonality (1.2%), and biotic soil parameters (0.4%). Different combinations of the predictors would explain the remaining variation, notably spatial distance/abiotic soil parameters (3%) and abiotic/biotic soil parameters (2.3%).

### Edaphic Parameters Influencing Cercozoan Communities

Among soil abiotic factors, soil moisture was most important (ANOVA: *F*_1,163_ = 11.546, *p* < 0.001), followed by clay, organic C, pH, total N, and NO_3_^−^ content (Table 3). Biotic parameters (microbial biomass C and N contents, number of bacteria, litter, archaeal 16S, plant biomass and fungal PLFAs) explained a significant but lower amount of the variation in cercozoan beta diversity (Table 3). Using linear mixed effect models, we specified which abiotic and biotic factors affected the 12 most abundant cercozoan families (Supplementary Table S6). Two-thirds of the cercozoan/endomyxan families (8 of 12) were positively affected by soil moisture (*p* < 0.001), but not the Spongomonadidae, the Allapsidae, or the parasitic *Spongospora nasturtii* and *Polymyxa* lineages. The relative abundances of the testate amoebae, i.e., Euglyphidae and unclassified Euglyphida, Trinematidae, and Rhogostomidae, responded negatively to soil moisture. In contrast, naked flagellates and amoeboflagellates were positively correlated with increasing soil moisture. Flagellates correlated negatively and amoeboflagellates positively with clay content. Flagellates, Sandonidae, and plant parasitic *Polymyxa* were positively correlated with pH, while testate amoebae (Euglyphidae) were negatively associated with pH (Supplementary Table S6).

**Table 3:** ANOVA results of the most parsimonious RDA model including selected edaphic and biotic parameters.

Best abiotic predictors	*F* ratio_1,163_	*p* value
Soil moisture	11.546	0.001
% of clay	4.8303	0.001
Soil organic C	3.7957	0.001
pH	3.3318	0.001
Total N	2.1179	0.001
NO_3_	1.929	0.007

**Best biotic predictors**	***F* ratio_1,168_**	***p* value**

N microbial biomass	6.9191	0.001
Number of bacteria	4.1349	0.001
Litter	2.361	0.001
Archaeal 16S	1.7796	0.004
Total plant biomass	1.6192	0.004
Fungal PLFAs	1.5051	0.024
C microbial biomass	1.4467	0.033

## Discussion

### Environmental Selection or Random Distribution?

Using Cercozoa and Endomyxa as models of very diverse and abundant soil protists, we demonstrated that their community composition in a small grassland site was non-random, but instead was spatially and temporally structured. With on average an estimated 1000 OTUs per gram of soil, high local species richness was a striking characteristic of cercozoan communities, with however small changes in beta diversity across space and time (Supplementary Table S5). Our results align with the consistent patterns of high alpha – and low beta diversity which have been found for protists in grasslands (Fiore-Donno et al., 2016) and, including specifically Cercozoa, in tropical forests (Lentendu et al., 2018). Sufficient sampling, attested by our saturation curves (Supplementary Figure S4), allowed us to further partition the beta diversity suggesting that deterministic processes drive the observed cercozoan community assembly. Nonetheless, our design did not allow us to exclude the influence of neutral dynamics and competitive interactions that may lead to similar distribution patterns (Dini-Andreote et al., 2015). Spatial distance (4.4%) and soil biotic and abiotic factors (2.9%) explained substantial variation indicating that cercozoan communities were significantly influenced by spatial gradients in the edaphic parameters (Figure 5 and Supplementary Figure S2).

On a small spatial scale, our results correspond to large-scale patterns of protistan distribution as described by Lentendu et al. (2018), who established environmental selection as the main process driving protistan spatial patterns. This is in striking contrast to two recent studies. Bahram et al. (2016) reported a random spatial distribution of small soil eukaryotes, including protists, in boreal forests at a range of 0.01 to 64 m. They explained the observed distribution by invoking drift and homogenizing dispersal. The most striking difference compared with our study is the low efficiency of their ITS2 primers for the retrieval of protists (66 rhizarian OTUs, including the Cercozoa). This is at least one order of magnitude lower than in our data, possibly indicating a non-thorough sampling of the rhizarian communities in their study, which could in turn have hampered a robust assessment of the observed distribution patterns. In the second study, Zinger et al. (2018) suggested the absence of dispersal limitation and a stochastic distribution of protists in a tropical forest, using a 10 m-resolution sampling grid. This might have been too coarse to detect spatial patterns of protists: according to our results, only negative correlations could be detected at such a distance (Figure 3).

Although we established the importance of environmental selection, homogenizing processes such as neutral assembly mechanisms may have contributed to the observed community assembly, as suggested by the positive autocorrelation of cercozoan communities up to a distance of 3.9 m (Figure 3). However, 18.32% of explained variance (Figure 5) highlighted the importance of microhabitats for the non-random distribution of the cercozoan communities.

### Environmental Selection and Patch Dynamics Selected for Specific Functional Traits

**FIGURE 6:**
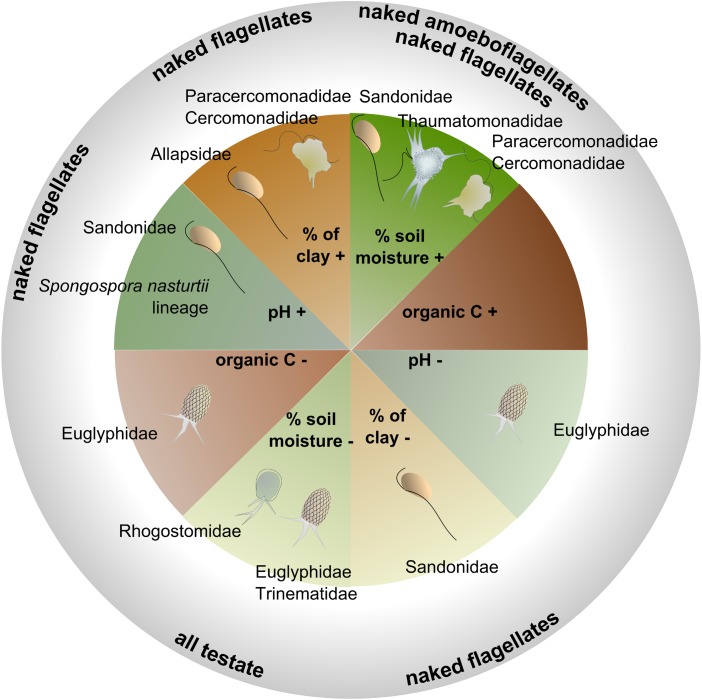
Schematic illustration showing the positive (+) or negative (–) interactions between the four most influential soil physicochemical parameters (based on the ANOVA, Table 3), and the relative abundances of the major families or morphotypes (details of the models in Supplementary Table S6).

Significant relationships between functional traits and soil abiotic and biotic factors (Supplementary Table S6) indicated that the distribution of protistan traits was driven by environmental selection in our study site. Some of these environmental filters showed marked spatial gradients (clay content, pH, total N), while other showed more temporal variation (moisture, NH_4_^+^, total plant biomass) (Supplementary Figure S2). Soil moisture is well known to influence microbial activity (Tecon and Or, 2017). The spatial variation in clay content together with the temporal variation in soil moisture triggered opposite responses from specific lineages or functional groups (Supplementary Table S6 and Figure 6).

Soil moisture was a major abiotic predictor in our study site, although with different effects according to taxonomic and functional groups. While flagellates and amoeboflagellates were favored by moisture, the cercozoan testate cells were correlated with drier conditions, suggesting patch dynamic processes connected to different living modes. In accordance with our model for Euglyphidae, Ehrmann et al. (2012) reported a preference of testate amoebae for relatively dry soils and low pH in forests. We can conclude that in grasslands, testate amoebae exhibit drought resistance, in contrast with naked cells and with cells covered with scales (i.e., Thaumatomonadidae), suggesting a protective role of the shell (Supplementary Table S4). Building a shell, however, slows down the reproduction rate (Schönborn, 1992), and thus generates a trade-off between protection and reproductive fitness. In contrast, amoeboflagellates and flagellates, with their faster reproduction rate (Ekelund and Rønn, 1994), would have an increased fitness in moist conditions.

The spatial distribution of soil clay content was an important structuring factor in our study site. Experimentally increasing clay content has been shown to improve water retention capacity and favor soil bacteria (Heijnen et al., 1993). In addition, it leads to a reduction of habitable pore space, which in our study seemed to favor the amoeboflagellates (Paracercomonadidae and Cercomonadidae) and the naked flagellates (Allapsidae, but not Sandonidae) (Figure 6 and Supplementary Table S6). The small percentage of free-swimming protists was negatively correlated with bulk density (Supplementary Table S6), suggesting their preference for larger soil pores. Another important structuring factor was pH, despite its low variability in our study site (Supplementary Figure S2 and Supplementary Table S1 – pH 6.08–7.23). Globally, cercozoans have been shown to prefer neutral or basic soils (Dupont et al., 2016) and their relative abundance was seen to increase significantly along a pH gradient from c. 4 to 6.5 (Shen et al., 2014). Over a narrower gradient, we showed that Sandonidae and the endomyxan parasitic *Spongospora nasturtii* lineage were positively correlated with a slightly basic pH, and the Euglyphidae with a slightly more acidic one (Figure 6 and Supplementary Table S6). Our results thus suggest that different taxa could have different pH preferences. Soil organic carbon had a negative effect only on the Euglyphidae, in conjunction with nitrogen content, while the C/N ratio had a positive effect, suggesting their preference for low-nutrient soils with a relatively higher C than N content.

Bacterial cell counts were a major explanatory biotic factor, negatively correlated with endomyxan plant parasites. This correlation was robust, also when taxonomy, nutrition or locomotion modes, and morphology, were considered. This illustrates the fundamental role of biotic interactions shaping microbial community structure and ultimately plant health (Hassani et al., 2018) and confirms the filtering effect on the cercozoan/endomyxan community structure revealed in the rhizosphere of *Arabidopsis thaliana* (Sapp et al., 2018). In our study, cercozoan taxa that positively correlated with bacterial numbers were identified as creeping/gliding on substrate. This is in accordance with soil protists feeding mostly on bacterial biofilms (Böhme et al., 2009). Interestingly, there was no positive correlation between bacterial cell counts and bacterivores. Most bacterivorous families, however, were strongly and positively correlated with soil moisture (Supplementary Table S6), which shared a strong peak in April with bacterial cell counts, potentially masking the effect of bacterial cell numbers (although there was no co-correlation between those variables across the year). Contrary to other bacterivores, Spongomonadidae did not follow the seasonal variation of the bacteria (Figure 4). This may be explained by their living modes, mostly in substrate-attached colonies (Strüder-Kypke and Hausmann, 1998). It has been suggested that the colonial species may also feed by saprotrophy (Strüder-Kypke and Hausmann, 1998), a hypothesis supported by our observed positive correlation with extractable organic carbon (Supplementary Table S6). We know very little about the interactions of archaea with soil protists. Thus it is worth pointing out the to date unexplained significant negative effect of archaeal abundance on Paracercomonadidae (Supplementary Table S6).

### Seasonal Variability Affected the Trophic Structure of Cercozoan Communities

Cercozoan communities showed seasonal oscillations, in accordance with results from bacteria in grasslands (Muller Barboza et al., 2018). The most significant community turnover occurred between April and May (Supplementary Figure S3A), when a series of concomitant changes in edaphic factors was observed (Supplementary Figure S2). The high soil moisture in April favored bacterial activity and proliferation. At the same time, high levels of extractable organic C indicated abundant root exudates or decomposition at the onset of the plant growing season, when plant total biomass was still low. We hypothesized that this triggered a series of events: the bacteria, usually C limited, were stimulated by this C input. Since fungi were not yet abundant, bacteria were mostly responsible for the high nitrogen content in the microbial biomass. Consequently, the release of NH_4_^+^ (also peaking in April) may be best explained by protistan predation on bacteria (Bonkowski, 2004). This was confirmed by the high abundances of five bacterivorous families (Figure 4). In May, bacteria, bacterivores, and nitrogen-related parameters all decreased, together with soil moisture. In sharp contrast, all three omnivorous families increased in May. We hypothesized that predation by omnivores could have contributed to the decline of the bacterivores, in addition to the negative effect of declining soil moisture (Supplementary Figure S2). Our study, based on DNA, does not make it possible to distinguish between active and resting stages, but we probably also amplified extracellular DNA from recently deceased cells. Thus, the seasonal variation we observed was probably an underestimate.

Our hypothesis that the abundance of plant parasites would follow the annual plant cycle was not supported, since they showed no seasonal variation (Supplementary Figure S2). Phytomyxeans are known to form resistant cysts in plant root hairs that remain for years in the soil after plant decay (Dixon, 2009).

### Number of OTUs, Diversity, and Functional Traits

Cercozoan diversity was in accordance with previous studies, which established Sarcomonadea (Glissomononadia and Cercomonadida) as the dominant class in different terrestrial habitats (Howe et al., 2009; Geisen et al., 2015; Fiore-Donno et al., 2018): more specifically in feces (Bass et al., 2016), in the soil of neotropical forests (Lentendu et al., 2018), on the leaves of Brassicaceae (Ploch et al., 2016), in heathlands (Bugge Harder et al., 2016), and in German grasslands, including the site studied here (Venter et al., 2017). Especially the glissomonads, (mostly) bacterivorous small flagellates, appear to dominate in all types of grasslands, where they can reach 5% of all protistan sequences (Geisen et al., 2015). However, the dominance of the remaining taxa is more variable between habitats. The Sarcomonadea were followed by the (mostly) omnivorous amoeboflagellates in cercomonads and by Cryomonadida, composed of amoebae or amoeboflagellates with organic, transparent tests. The widespread presence of Cryomonadida in soil has been overlooked in observation-based inventories, but confirmed by molecular environmental sampling (Lentendu et al., 2014; Bass et al., 2016; Bugge Harder et al., 2016; Ploch et al., 2016); especially the genus *Rhogostoma* is very common in soil (Fiore-Donno et al., 2018).

In conclusion, we showed that environmental selection driven by abiotic and biotic edaphic factors significantly determined community assembly of Cercozoa and Endomyxa. Considering functional traits and their trade-offs, we were able to highlight the importance of environmental selection and patch dynamics as underlying processes. We believe that our study has bearing for other soil protists and soil ecosystems beyond the limits of this small grassland plot. Once the patterns underlying the small-scale distribution of protists are detected, they can be upscaled and contribute to understanding global protistan biogeographies. This is a perequisite for predicting effects of human-induced changes (i.e., land management or global warming) on these widespread and functionally important soil organisms.

## Author Contributions

EK and SM developed the design of the SCALEMIC experiment. KMR performed the DNA extractions. KMR and RSB analyzed the soil properties. AMF-D conducted the amplifications, Illumina sequencing, and bioinformatics pipeline. TR-H, FD, and AMF-D performed the statistics. AMF-D, TR-H, and MB interpreted the data. AMF-D wrote the manuscript. All authors participated in the revisions and approved the final version of the manuscript.

## Conflict of Interest Statement

The authors declare that the research was conducted in the absence of any commercial or financial relationships that could be construed as a potential conflict of interest. The reviewer, BF, declared a past co-authorship, with several of the authors, AMF-D, KD, and MB, to the handling Editor.

## Footnotes

1 http://sankey-diagram-generator.acquireprocure.com/ (accessed October 2018).
