# Erratum: Functional Traits and Spatio-Temporal Structure of a Major Group of Soil Protists (Rhizaria: Cercozoa) in a Temperate Grassland

**DOI:** 10.3389/fmicb.2019.01654

**Published:** 2019-07-16

**Authors:** Frontiers Production Office

**Affiliations:** Frontiers Media SA, Lausanne, Switzerland

**Keywords:** biogeography, functional traits, soil ecology, protozoa, microbial assembly, environmental selection, dispersal limitation, soil protists

Due to an editorial error, the Data Availability Statement containing the raw sequences was erroneously omitted from the article. The publisher apologizes for the mistake.

## Data Availability

Raw sequences have been deposited in Sequence Read Archive (SRA, NCBI) SRR6187016 and BioProject PRJNA414535; the 694 OTUs (representative sequences) under GenBank accession # MG242652-MG243345.

