# Supplementary material for: Functional Traits and Spatio-Temporal Structure of a Major Group of Soil Protists (Rhizaria: Cercozoa) in a Temperate Grassland
Source: Front Microbiol. 2019 Jun 11;10:1332. doi: 10.3389/fmicb.2019.01332 (PMC6579879; doi:10.3389/fmicb.2019.01332)
Supplement: Supplementary file 1 [file Data_Sheet_1.zip › Data Sheet 1/FioreDonnoSupplMat/FigS1SampleDesign.pdf]

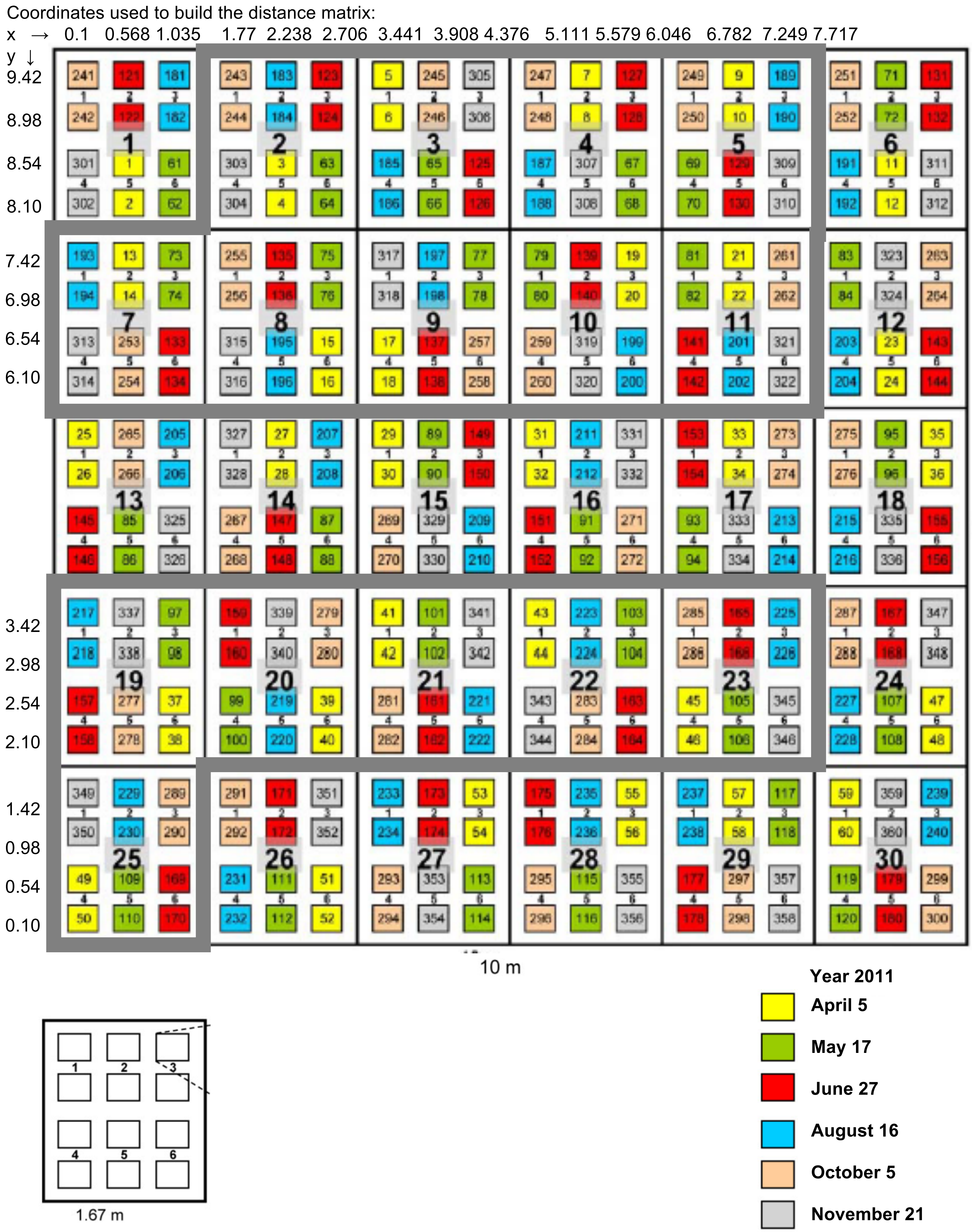

Figure S1. Sampling design of the 10m^2 grassland study site (Fig. A2 in Regan et al. 2014), with the 360 samples collected at six different dates and the spatial coordinates used to build the distance matrix.
For this study, we selected the samples from the 15 areas outlined in grey (12 samples/area = 180 samples).
The DNA from samples 125, 185 and 305 from area 3 could not be amplified.
