# Supplementary material for: Functional Traits and Spatio-Temporal Structure of a Major Group of Soil Protists (Rhizaria: Cercozoa) in a Temperate Grassland
Source: Front Microbiol. 2019 Jun 11;10:1332. doi: 10.3389/fmicb.2019.01332 (PMC6579879; doi:10.3389/fmicb.2019.01332)
Supplement: Supplementary file 1 [file Data_Sheet_1.zip › Data Sheet 1/FioreDonnoSupplMat/FigS3PCoA.pdf]

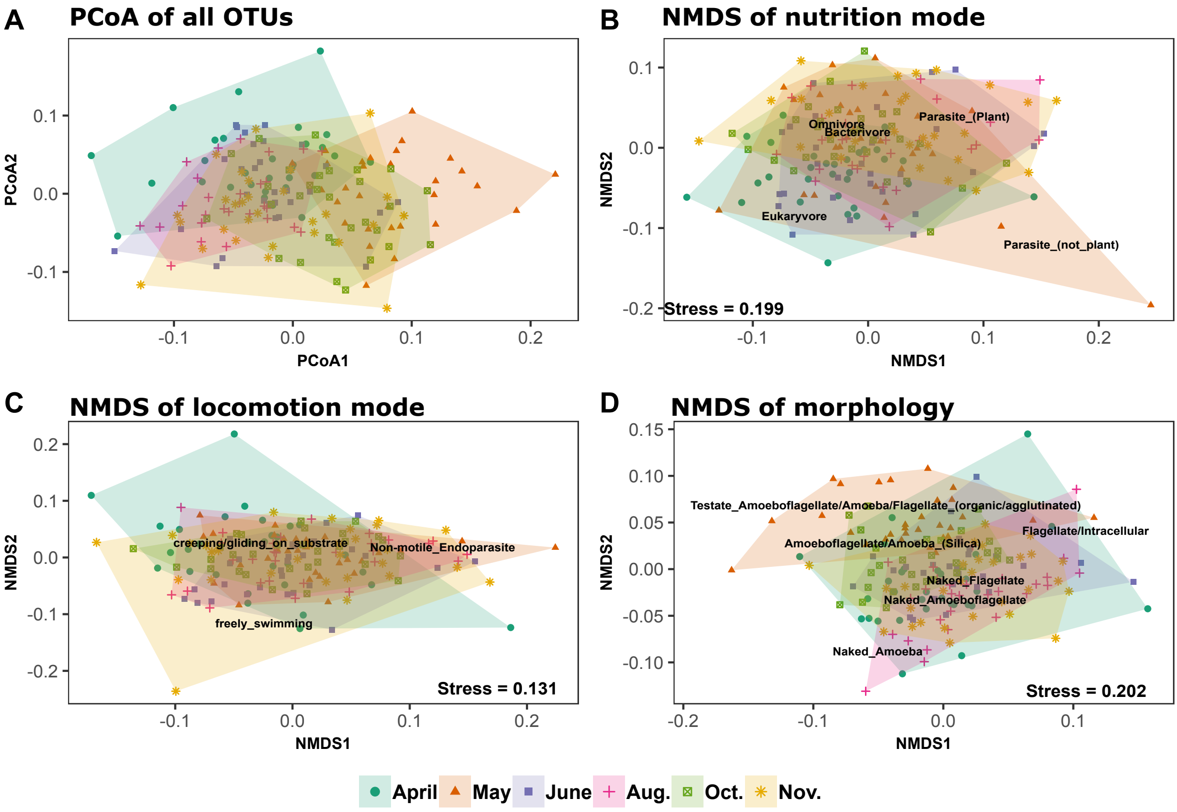

Figure S3. Ordination pots colored by sampling season. **A.** Principal Coordinate Analysis ordination (PCoA) of Bray-Curtis dissimilarities of the OTUs, by sampling sites; only April and May form distinct clusters. **B-D.** Non-Metric Multidimensional Scaling (NMDS) biplot of Bray-Curtis dissimilarities of the functional traits. All abundance tables were scaled to total sums before calculating the dissimilarities.
