# Supplementary material for: Functional Traits and Spatio-Temporal Structure of a Major Group of Soil Protists (Rhizaria: Cercozoa) in a Temperate Grassland
Source: Front Microbiol. 2019 Jun 11;10:1332. doi: 10.3389/fmicb.2019.01332 (PMC6579879; doi:10.3389/fmicb.2019.01332)
Supplement: Supplementary file 1 [file Data_Sheet_1.zip › Data Sheet 1/FioreDonnoSupplMat/FigS6Rarefaction.pdf]

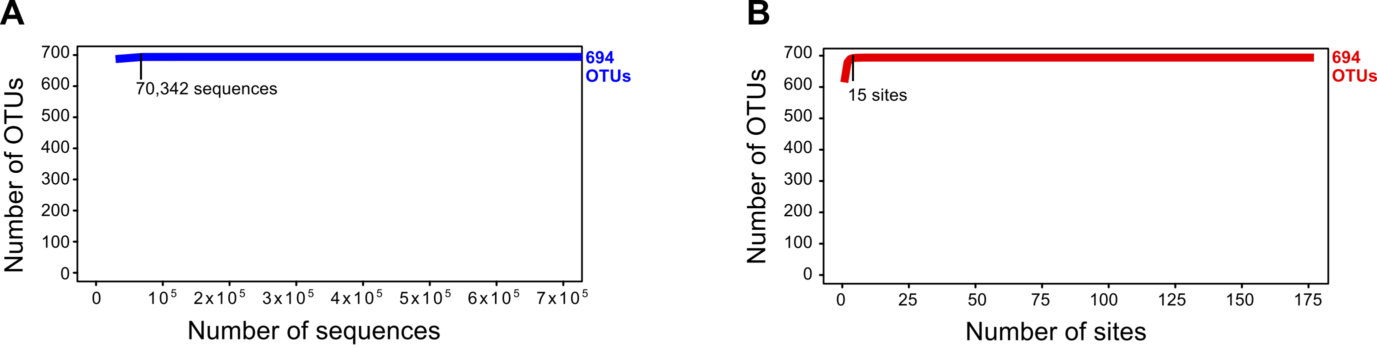

Figure S6. Description of the diversity. A. Rarefaction curve describing the observed number of OTUs as a function of the sequencing effort; saturation was reached with c. 70000 sequences.

B. Species accumulation curve describing the sampling effort; saturation was reached with 15 samples.
