# Supplementary material for: Functional Traits and Spatio-Temporal Structure of a Major Group of Soil Protists (Rhizaria: Cercozoa) in a Temperate Grassland
Source: Front Microbiol. 2019 Jun 11;10:1332. doi: 10.3389/fmicb.2019.01332 (PMC6579879; doi:10.3389/fmicb.2019.01332)
Supplement: Supplementary file 1 [file Data_Sheet_1.zip › Data Sheet 1/FioreDonnoSupplMat/SupplData.pdf]

Supplementary Data 1. Detailed description of the statistical and phylogenetic analyses

All statistical analyses were carried out within the R environment (R v. 3.5.1) (R Development Core Team, 2014). Analyses were performed with the package vegan (Oksanen et al., 2013), if not stated otherwise.

To evaluate if more sampling and sequencing effort would have revealed more richness, we carried out a rarefaction analysis based on OTUs accumulation curves, function *specaccum*, method rarefaction and 1000 random permutations; species richness was extrapolated using the function *specpool*. Indices of alpha-diversity, i.e. species richness and linearized Simpson diversity (Hill numbers ^{0}D and ^{2}D, respectively), were computed from the raw OTU table (Table S3) using the function *iNEXT* for each season, with default settings (50 bootstraps, confidence interval 0.95) at a common sample size of twice the smallest sample in the dataset (Chao et al., 2014).

Beta diversity was calculated as multiple site dissimilarities for each sampling date and for functional groups with functions *beta.multi* (presence-absence data, Sorensen index); with *beta.multi.abund* (abundance data, Bray-Curtis dissimilarities) for the total dissimilarities weighted by abundance; and with *beta.sample.abund*, which resamples Bray-Curtis dissimilarities from random subsets of 20 sites per sampling date with 100 permutations (abundance data) - all functions from package betapart (Baselga, 2017).

1.1.1 Spatial analyses

The effect of distance on beta diversity was assessed using a Mantel test with 9999 permutations using the Spearman correlation coefficient (function *mantel*) between the Bray-Curtis dissimilarities matrix described above (Hellinger-transformed with the function *decostand*) and the Euclidian distance matrix based on sample coordinates (function *dist*, Figure S1). We considered all OTUs, and OTUs grouped by the most represented functional classes and by seasons. To identify at which distances spatial correlations were found, we calculated Mantel correlograms using the Spearman correlation coefficient (function *mantel.corelog*, 9999 permutations) and definite break points. We considered break points to approximate distances of 0.25, 0.5 and 1m. Since it is important to obtain classes with same order of magnitude, the largest distance classes (8 to 12 m - with 10^2 instead of 10^3 pairwise distances) were binned together.

1.1.2 Seasonal variation

To test whether seasonal variation affected beta diversity, we performed multiple tests using the normalized OTU table: analysis of similarity (anosim), multi-response permutation procedure (MRPP) and multivariate permutational analysis of variance (PERMANOVA), all carried out with 999 permutations (functions *anosim*, *mrpp*, *adonis2*, respectively). To visualize how the seasonal variation affected the beta-diversity, we conducted non-metric multidimensional scaling (NMDS) and Principal Coordinates Analysis (PCoA) using the Bray Curtis distance matrix (scaled to the total sum); NMDS was used for trait data (function *metaMDS*); PCoA for all OTUs (function *cmdscale*) since NMDS failed to converge. These results are presented as graphs (Fig. S3). The response of different taxonomic groups to seasonal change was examined with

estimated marginal means (EMM, function *emmeans*, package emmeans) (Lenth, 2018) on general least square models (GLS, function *gls*, package nlme) (Pinheiro et al., 2018). Correction for spatial autocorrelation was done by selecting the best match out of four different correlation structures (spherical, gaussian, rational, exponential) based on the Akaike Information Criterion (AIC). The best model, i.e. with the lowest AIC, was then used to obtain EMM-based contrasts between sampling dates.

1.1.3 Environmental variables

The influence of environmental variables on specific groups of cercozoans (normalized abundances) was assessed using general linear mixed models (GLMM) where sampling date was introduced as a mixed random effect (function *lme*, package nlme) to account for seasonal change. As for the GLS models described above, correction for spatial autocorrelation was done by selecting the best model based on the lowest AIC value. Variable selection was carried out as follows: the best environmental predictors were preselected using the forward and backward selection approach (function *stepAIC*, package MASS) (Venables and Ripley, 2002). The set of candidate predictors was subsequently used for multi-model inference, based on the corrected AIC (AICc) (function *dredge*, package MuMIn) (Barton, 2018). This procedure yielded a list of potential models ranked by AICc. From this list, we considered a variable as potentially important if it appeared in all models within an interval of two units of AICc from the best scoring model (function *model.avg*, package MuMIn). In a final step to obtain even more sparse models, these “consensus” variables were only selected if they were found to be highly significant ($p<0.01$) in a final GLMM call. R^2 was calculated with the function *r.squaredGLMM* in the package MuMIn.

1.1.4 Variance partitioning

Variance partitioning among biotic and biotic edaphic parameters, spatial and temporal components was assessed using the function *varpart* and visualized in a Venn diagram. Prior to the analysis, the OTU table was Hellinger-transformed (function *decostand*) and environmental data were normalized. Spatial and temporal structures in the response data (OTUs) were previously detected using Principal Coordinates of Neighbor Matrices (PCNM) (Borcard and Legendre, 2002; Borcard et al., 2004; Dray et al., 2006). Briefly, an Euclidean distance matrix was calculated from spatial and temporal data and truncated to obtain a new set of independent spatial and temporal PCNM variables (neighbor matrix). For each set of PCNM variables, a principal coordinate analysis (*cmdscale* function) was carried out to select those PCNMs with positive eigenvalues. The resulting sets of PCNM variables were used in a redundancy analysis (function *rda*) with the *nondetrended* response data. The significant spatial and temporal PCNM variables were selected using the *forward.sel* function and the resulting model was assessed using the *anova* function. Constrained analysis was performed similarly, exept that the significant environmental variables were selected using the *ordiR2step* function. The resulting parsimonious model was tested using the *anova* function and the significance of each parameter was determined using the anova function *by term*. Variance partitioning (Peres-Neto et al., 2006) among the significant PCNM (spatial and temporal) and biotic and biotic edaphic variables was carried out using the function *varpart*.

1.1.5 Phylogenetic analyses

The 694 OTUs obtained were added to a published reference alignment of nearly complete 18S sequences of 176 cercozoan taxa (Fiore-Donno et al., 2018). The alignment had 1447 positions, 1013 alignment patterns, and a proportion of undetermined characters of 66.28%. Maximum likelihood (ML) analyses were run using RAxML 8.2.4 (Stamatakis, 2014) with the GTR model of substitution and a 25 rate category discrete gamma distribution. The best scoring ML tree was inferred from 200 randomized starting Maximum Parsimony trees using the GTRMIX model. The best-scoring tree was used to report the confidence values as percentages obtained through 500 non-parametric bootstraps.

References

Barton, K. (2018). Multi-Model Inference. Retrieved from https://CRAN.R-project.org/package=MuMIn.

Baselga, A. (2017). Partitioning abundance-based multiple-site dissimilarity into components: balanced variation in abundance and abundance gradients. *Methods in Ecology and Evolution* 8(7), 799-808. doi: 10.1111/2041-210X.12693.

Borcard, D., and Legendre, P. (2002). All-scale spatial analysis of ecological data by means of principal coordinates of neighbour matrices. *Ecological Modelling* 153, 51-68. doi: 10.1016/S0304-3800(01)00501-4.

Borcard, D., Legendre, P., Avois-Jacquet, C., and Tuomisto, H. (2004). Dissecting the spatial structures of ecological data at all scales. *Ecology* 85, 1826-1832. doi: 10.1890/03-3111.

Chao, A., Gotelli, N.J., Hsieh, T.C., Sander, E.L., Ma, K.H., Colwell, R.K., et al. (2014). Rarefaction and extrapolation with Hill numbers: a framework for sampling and estimation in species diversity studies. *Ecological Monographs* 84, 45-67. doi: 10.1890/13-0133.1.

Dray, S., Legendre, P., and Peres-Neto, P.R. (2006). Spatial modelling: a comprehensive framework for principal coordinate analysis of neighbour matrices (PCNM). *Ecological Modelling* 196, 483-493. doi: 10.1016/j.ecolmodel.2006.02.015.

Fiore-Donno, A.M., Rixen, C., Rippin, M., Glaser, K., Samolov, E., Karsten, U., et al. (2018). New barcoded primers for efficient retrieval of cercozoan sequences in high-throughput environmental diversity surveys, with emphasis on worldwide biological soil crusts. *Molecular Ecology Resources* 18(2), 229-239. doi: 10.1111/1755-0998.12729.

Lenth, R. (2018). emmeans: Estimated Marginal Means, aka Least-Squares Means. Retrieved from https://CRAN.R-project.org/package=emmeans.

Oksanen, J., Blanchet, F.G., Kindt, R., Legendre, P., Minchin, P.R., O'Hara, R.B., et al. (2013). Vegan: Community Ecology Package. R package version 2.0-10. http://CRAN.R-project.org/package=vegan.

Peres-Neto, P.R., Legendre, P., Dray, S., and Borcard, D. (2006). Variation partitioning of species data matrices: estimation and comparison of fractions. *Ecology* 87, 2614-2625. doi: 10.1890/0012-9658(2006)87.

Pinheiro, J., Bates, D., DebRoy, S., Sarkar, D., and Team, R.D.C. (2018). nlme: Linear and Nonlinear Mixed Effects Models, R package version 3.1-137. Retrieved from http://CRAN.R-project.org/package=nlme.

R Development Core Team (2014). "R: A language and environment for statistical computing", (ed.) R.F.f.S. Computing. (Vienna, Austria).

Stamatakis, A. (2014). RAxML version 8: a tool for phylogenetic analysis and post-analysis of large phylogenies. *Bioinformatics* 30(9), 1312-1313. doi: 10.1093/bioinformatics/btu033.

Venables, W.N., and Ripley, B.D. (2002). *Modern applied statistics with S.* New York: Springer.
