# Supplementary material for: Functional Traits and Spatio-Temporal Structure of a Major Group of Soil Protists (Rhizaria: Cercozoa) in a Temperate Grassland
Source: Front Microbiol. 2019 Jun 11;10:1332. doi: 10.3389/fmicb.2019.01332 (PMC6579879; doi:10.3389/fmicb.2019.01332)
Supplement: Supplementary file 1 [file Data_Sheet_1.zip › Data Sheet 1/FioreDonnoSupplMat/SupplLegends.pdf]

Fiore-Donno et al. 2019, Community structure of soil protists (Cercozoa)

Supplementary information, legends

Supplementary Data 1. Detailed description of the statistical and phylogenetic analyses.

Supplementary tables

Table S1. Environmental parameters from the study site as in Regan *et al.* (2014) used in our statistical analyses. Their seasonal variation is shown in Fig. S2.

Table S2. Combinations of barcodes used in this study, with the corresponding samples.

Table S3. Database of the abundance of each OTU per sample. The taxonomic assignment (supergroup, class, order, family, genus and species) is provided according to the best hit by BLAST (PR2 database), with the % of similarity. Functional traits (morphology, nutrition and locomotion modes) were estimated following Table S4.

Table S4. References for the functional traits of the cercozoan/endomyxan taxa identified in this study.

Table S5. Beta diversity indices calculated for each sampling date.

Table S6. Linear mixed models showing the effects of the environmental predictors on the most abundant 12 cercozoan/endomyxan families, the morphotype, nutrition and locomotion modes. We give: a) the spatial correlation structure best correcting the starting model according to the AIC; b) the number of models within two AICc units (after model dredging); c) the number of predictors included in all models extracted in a); d) the remaining, highly significant predictors after fitting a model with just the consensus predictors in b), and their effect type (positive or negative); e) their significance level (p values: *<0.05, **<0.01, ***<0.001). f) the R^2 of the fixed effects of the final model with only the highly significant predictors (d); g) the R^2 of the fixed and random effects of the final model with only the highly significant predictors.

Supplementary figures

Figure S1. Sampling design of the $10m^2$ grassland study site (Fig. A2 in Regan *et al*., 2014), with the 360 samples collected at six different dates and the spatial coordinates used to build the distance matrix. For this study, we selected the samples from the 15 areas outlined in grey (12 samples/area=180 samples). The DNA from samples 125, 185 and 305 from area 3 could not be amplified.

Figure S2. Box plots showing the seasonal variation of the environmental parameters from Table S1.

Figure S3. Ordination plots colored by sampling season. **A.** Principal Coordinate Analysis ordination (PCoA) of Bray-Curtis dissimilarities of the OTUs, by sampling sites; only April and May form distinct clusters. **B-D**. Non-Metric Multidimensional Scaling (NMDS) biplot of Bray-Curtis dissimilarities of the functional traits. All abundance tables were scaled to total sums before calculating the dissimilarities.

Figure S4. Similarities of the OTUs with known sequences. OTUs are classified according to their percentage of similarity to the next kin by BLAST. The horizontal bar length is proportional to the number of OTUs in each rank. Shaded area=OTUs with a similarity ≥ 97%.

Figure S5. Cercozoa/Endomyxa Maximum Likelihood phylogenetic tree, inferred from an alignment of 870 taxa and 1,447 positions. The tree is rooted between Phytomyxea and the remaining taxa. OTUs found in this study are in bold. Main clades are named according to the PR2 database taxonomy, clades for which no OTUs were found are named in gray. Bootstrap values are given for each node. The scale bar indicates the fraction of substitutions per site.

Figure S6. Description of the diversity. **A**. Rarefaction curve describing the observed number of OTUs as a function of the sequencing effort; saturation was reached with c. 70000 sequences. **B**. Species accumulation curve describing the sampling effort; saturation was reached with 15 samples.
