# Supplementary material for: Functional Traits and Spatio-Temporal Structure of a Major Group of Soil Protists (Rhizaria: Cercozoa) in a Temperate Grassland
Source: Front Microbiol. 2019 Jun 11;10:1332. doi: 10.3389/fmicb.2019.01332 (PMC6579879; doi:10.3389/fmicb.2019.01332)
Supplement: Supplementary file 1 [file Data_Sheet_1.zip › Data Sheet 1/FioreDonnoSupplMat/TableS1EnvPar.pdf]

Table S1. Environmental parameters from the study site as in Regan *et al.*, (2014) used in our statistical analyses. Their seasonal variation is shown in Figure S2.

	Soil_moisture	Bulk_density	Clay_percent	Roots	pH	Total_N	Organic_C	CN_ratio	NH_4^+	NO_3^-	PO_4^{2-}	C_microbial_biomass	N_microbial_biomass	Extractable_Organic_C	Extractable_Organic_N	Fungal_PLFAs	Number_of_bacteria	Bacterial_16S	Archaeal_16S	Plant_litter_biomass	Total_plant_biomass
Unit	%	g cm^{-3}	% relative to silt	g/soil core		µg*g-1 soil dw	µg*g-1 soil dw		µg*g-1 soil dw	µg*g-1 soil dw	µg*g-1 soil dw	µg*g-1 soil dw	µg*g-1 soil dw	µg*g-1 soil dw	µg*g-1 soil dw	µg g-1 dw	cells g-1 dw	number of copies / g dw	number of copies / g dw	g 20cm2	g 20cm2
S003	63.20	0.83	16.47	2.37	6.13	0.63	6.47	10.19	13.56	24.13	88.46	1679.34	262.90	231.08	11.99	1.54	5.64E+10	3.96E+11	1.14E+09	4.98	11.28
S004	54.50	0.79	16.06	0.87	6.49	0.56	5.51	9.84	12.36	6.59	69.07	1464.90	213.15	212.85	18.09	1.62	2.30E+10	3.33E+11	1.94E+09	5.95	9.08
S005	56.70	0.75	18.23	3.00	6.55	0.65	6.49	10.00	12.06	29.20	85.71	1787.47	271.40	200.78	8.72	1.81	3.16E+10	4.06E+11	3.55E+09	2.95	8.70
S006	59.15	0.80	17.70	3.48	6.30	0.64	6.52	10.18	14.66	22.36	83.17	1672.75	251.27	199.55	9.04	1.83	3.13E+10	3.24E+11	2.48E+09	6.61	10.89
S007	61.24	0.77	18.10	1.22	6.52	0.68	6.89	10.20	15.13	7.09	75.90	1952.62	295.48	220.93	19.59	1.81	2.34E+10	4.24E+11	2.89E+09	3.85	8.78
S008	60.03	0.81	17.05	3.03	6.82	0.67	6.84	10.21	15.62	23.83	88.37	1874.11	293.56	220.85	10.17	1.65	2.45E+10	3.89E+11	2.22E+09	5.62	9.00
S009	61.99	0.87	16.95	1.29	6.36	0.66	6.66	10.14	16.51	22.11	72.30	1728.85	260.16	181.07	6.95	1.98	2.39E+10	2.12E+11	3.79E+09	4.72	10.38
S010	57.33	0.74	15.70	0.90	6.57	0.68	7.16	10.59	16.87	7.70	67.68	1910.61	281.67	205.27	13.90	2.02	1.82E+10	2.99E+11	2.80E+09	5.30	8.17
S013	59.13	0.54	14.61	1.54	6.76	0.69	7.07	10.19	18.32	15.21	85.41	1790.22	311.66	253.52	11.86	1.69	4.54E+10	4.61E+11	5.53E+09	2.25	4.39
S014	54.05	0.95	15.62	1.46	6.84	0.64	6.45	10.13	13.05	24.60	93.34	1653.95	264.78	213.66	5.67	1.60	5.42E+10	2.58E+11	2.19E+09	5.26	7.12
S015	58.75	0.98	14.67	1.62	6.56	0.63	6.32	10.11	15.91	17.61	88.30	1754.65	276.46	199.54	8.72	3.26	1.11E+10	2.43E+11	1.56E+09	7.29	9.93
S016	55.15	0.96	14.87	0.67	6.63	0.67	6.98	10.35	14.99	6.06	62.74	1755.28	257.57	212.57	16.38	2.17	3.07E+10	3.58E+11	3.49E+09	3.09	6.08
S017	56.93	0.98	14.77	1.31	6.63	0.65	6.70	10.27	19.49	24.64	67.28	1699.59	248.41	193.71	6.28	1.85	1.69E+10	3.62E+11	4.16E+07	9.20	12.17
S018	56.71	0.99	15.09	1.70	6.55	0.65	6.49	9.94	13.93	26.82	84.85	1752.26	276.27	174.50	4.30	1.42	1.90E+10	2.52E+11	5.75E+08	6.34	9.91
S019	60.13	0.92	16.08	1.53	6.52	0.64	6.45	10.00	29.10	11.61	79.12	1820.81	280.89	355.42	32.96	2.80	2.83E+10	3.67E+11	2.35E+09	3.29	8.90
S020	58.46	1.03	15.46	1.14	6.70	0.68	6.98	10.29	13.49	26.33	73.53	1636.69	248.00	179.65	4.27	1.59	1.09E+10	4.07E+11	1.76E+09	5.55	10.19
S021	55.72	0.97	16.20	1.08	6.51	0.67	6.69	10.03	15.48	15.15	63.55	1584.14	241.77	173.24	9.34	1.83	1.47E+10	3.78E+11	1.51E+09	5.12	7.96
S022	58.04	0.99	14.10	2.24	6.56	0.64	6.59	10.21	13.29	7.92	70.71	1605.58	246.04	191.18	13.96	1.79	3.80E+10	6.45E+11	2.15E+09	4.22	6.40
S037	57.95	0.87	12.58	2.14	7.02	0.67	6.78	10.13	17.43	7.25	101.64	1966.03	337.32	252.74	16.32	1.99	1.29E+10	5.30E+11	3.37E+09	2.46	3.47
S038	54.59	1.00	13.54	1.21	6.80	0.62	6.23	9.97	14.17	15.57	74.56	1674.44	245.30	191.04	6.94	1.65	1.57E+10	6.04E+11	2.17E+09	3.14	4.13
S039	55.12	0.80	12.23	2.91	6.57	0.68	6.91	10.11	28.69	67.66	128.05	1970.79	296.41	225.09	6.79	1.85	2.06E+10	2.81E+11	2.01E+09	2.10	3.43
S040	57.18	1.01	12.47	2.85	6.88	0.60	5.99	9.98	18.04	12.75	128.21	1814.46	278.28	237.06	16.62	2.36	2.93E+10	2.45E+11	1.76E+09	2.91	4.01
S041	57.45	1.04	15.05	1.98	6.75	0.66	6.55	10.00	12.12	20.79	87.29	1779.02	261.24	217.01	8.67	1.46	1.79E+10	3.02E+11	3.12E+09	3.86	6.20
S042	57.57	1.18	12.68	1.73	6.52	0.59	6.06	10.23	19.21	12.91	58.81	1456.60	215.44	187.22	9.43	1.39	1.61E+10	1.71E+11	1.68E+09	4.87	6.84
S043	57.98	1.03	13.29	1.18	6.81	0.65	6.47	9.93	15.85	6.34	73.10	1731.53	276.13	222.94	17.99	1.85	1.34E+10	6.77E+09	1.67E+09	4.39	6.15
S044	56.62	0.96	11.83	1.74	6.70	0.67	6.60	9.92	11.67	20.54	69.75	1602.51	226.28	169.39	5.51	1.60	1.59E+10	2.65E+11	6.29E+08	6.00	9.31
S045	57.19	1.03	12.32	1.20	6.58	0.66	6.79	10.29	17.37	19.80	83.94	1895.28	308.16	187.95	6.46	2.05	2.04E+10	3.19E+11	3.04E+09	0.80	2.70
S046	58.78	0.86	13.58	2.71	6.81	0.68	7.02	10.31	17.32	6.13	94.24	1761.92	296.48	259.85	18.11	1.75	1.19E+10	3.10E+11	1.56E+09	5.35	7.36
S049	55.00	0.99	15.13	4.07	6.55	0.57	5.74	10.06	14.41	4.74	57.87	1492.02	206.68	200.86	21.80	1.93	2.09E+10	2.79E+11	NA	4.97	8.73
S050	50.81	1.01	15.20	2.51	6.70	0.56	6.07	10.78	11.04	14.12	45.79	1343.29	191.38	141.72	3.57	1.23	1.15E+10	2.88E+11	1.70E+09	5.78	7.38
S063	26.15	0.75	16.47	2.53	6.21	0.64	6.51	10.17	7.41	7.95	59.22	1487.35	187.18	171.99	11.23	2.27	1.02E+10	1.42E+11	1.52E+09	2.14	14.20
S064	26.83	0.85	16.06	1.06	6.68	0.61	6.08	9.93	6.61	11.72	55.04	1362.62	179.87	115.91	3.65	1.54	7.37E+09	1.44E+11	2.92E+09	6.32	20.57
S065	26.77	0.91	18.23	1.43	6.53	0.63	6.31	9.96	5.07	9.69	72.74	1362.22	181.01	160.34	12.68	1.83	2.77E+09	1.54E+11	2.51E+09	2.66	7.31
S066	34.64	0.88	17.70	0.57	6.45	0.66	6.56	9.95	5.52	12.15	81.90	1714.88	252.90	165.02	14.35	3.24	8.04E+09	1.76E+11	2.76E+09	3.36	11.44
S067	29.43	0.84	18.10	0.66	6.65	0.64	6.47	10.12	6.74	14.44	62.78	1571.20	201.79	118.79	3.60	1.80	3.40E+09	1.72E+11	2.76E+09	4.68	12.62
S068	27.39	0.79	17.05	1.17	6.62	0.71	7.23	10.23	7.06	10.76	68.24	1554.34	222.01	152.72	9.78	3.60	5.08E+09	1.62E+11	3.02E+09	4.22	13.17
S069	29.73	0.76	16.95	2.32	6.46	0.67	6.66	9.97	5.30	7.99	76.48	1675.64	234.34	170.64	22.88	4.10	2.08E+09	1.77E+11	1.74E+09	2.42	12.91
S070	26.13	0.78	15.70	1.97	6.62	0.69	7.14	10.41	5.57	15.49	74.25	1898.60	226.91	156.48	8.10	2.04	3.81E+09	2.08E+11	3.13E+09	6.03	23.78
S073	26.40	0.89	14.61	1.49	6.74	0.58	5.86	10.09	5.77	11.59	61.77	1676.60	187.30	123.99	6.76	1.53	2.48E+09	1.58E+11	2.17E+09	2.88	7.68
S074	27.35	0.80	15.62	1.45	6.72	0.61	6.22	10.18	6.26	8.34	60.13	1468.12	206.80	156.43	10.50	2.18	2.82E+09	2.04E+11	2.42E+09	2.26	16.38
S075	29.64	0.92	14.67	0.74	6.50	0.64	6.47	10.09	5.37	7.80	73.10	1426.44	198.62	176.53	17.40	3.77	4.43E+09	1.40E+11	3.05E+09	1.39	9.88
S076	26.78	0.98	14.87	0.76	6.73	0.63	6.15	9.75	6.11	14.79	60.79	1386.20	182.36	115.04	4.18	1.38	2.37E+09	1.49E+11	3.19E+09	1.88	6.99

S077	29.24	0.87	14.77	0.88	6.58	0.61	5.96	9.83	5.68	10.31	57.39	1136.92	152.53	165.60	13.13	1.80	4.27E+09	1.63E+11	2.31E+09	4.71	10.10
S078	26.92	0.81	15.09	1.11	6.62	0.64	6.17	9.67	6.51	6.59	63.02	1640.88	242.95	168.23	13.00	2.51	5.70E+09	1.82E+11	2.58E+09	4.21	13.83
S079	25.58	0.86	16.08	1.59	6.51	0.56	5.65	10.15	6.20	2.16	46.04	1073.71	117.31	229.82	21.23	1.63	3.33E+09	1.05E+11	1.14E+09	9.67	27.61
S080	30.36	0.87	15.46	1.03	6.70	0.70	6.95	9.99	5.94	7.64	79.27	1613.10	239.07	161.18	19.98	2.73	2.20E+09	3.03E+11	2.32E+09	3.83	16.24
S081	27.34	0.81	16.20	1.64	6.70	0.67	6.86	10.17	5.98	8.81	51.90	1695.18	239.72	150.82	10.14	2.83	2.97E+09	2.22E+11	3.00E+09	3.83	14.72
S082	27.91	0.86	14.10	1.34	6.56	0.71	7.19	10.19	5.10	13.50	59.27	1591.73	204.17	121.09	5.44	1.88	2.90E+09	2.16E+11	3.23E+09	4.00	15.32
S097	30.08	0.74	12.58	1.60	6.83	0.67	6.58	9.90	4.30	18.72	71.90	1603.33	241.33	133.81	1.17	2.72	3.42E+09	2.13E+11	3.23E+09	2.67	8.50
S098	29.88	0.86	13.54	1.15	6.91	0.66	6.81	10.32	8.31	12.19	84.50	1416.28	212.51	181.19	9.48	2.33	1.75E+09	2.22E+11	3.14E+09	1.43	12.28
S099	26.48	0.83	12.23	1.46	6.99	0.67	7.39	11.07	7.76	9.04	92.35	1672.98	282.92	195.87	12.53	3.13	2.87E+09	1.33E+11	1.48E+09	0.96	8.26
S100	28.30	0.95	12.47	1.60	6.87	0.66	6.93	10.49	6.31	16.15	80.04	1535.31	222.92	144.25	5.19	2.37	2.86E+09	2.58E+11	3.55E+09	1.78	15.28
S101	27.86	0.71	15.05	1.07	6.58	0.64	6.37	9.88	6.13	9.09	67.19	1192.93	160.78	157.53	17.52	1.97	2.23E+09	2.30E+11	2.28E+09	3.70	12.44
S102	26.17	0.83	12.68	2.12	6.61	0.64	6.54	10.25	7.20	5.88	71.54	1682.17	206.23	200.22	17.82	3.43	2.95E+09	2.18E+11	3.76E+09	5.65	19.13
S103	27.21	0.86	13.29	1.05	6.79	0.72	7.41	10.25	4.26	16.91	73.18	1873.78	259.66	139.70	4.21	2.83	2.43E+09	2.45E+11	4.57E+09	3.34	16.16
S104	29.58	0.70	11.83	1.70	6.65	0.71	7.11	10.07	8.25	16.14	66.64	1461.45	236.95	148.38	7.25	2.54	3.02E+09	2.15E+11	4.01E+09	4.49	13.09
S105	30.72	0.84	12.32	1.96	6.67	0.64	6.51	10.11	6.81	11.86	68.04	1637.90	237.39	155.25	13.05	3.15	1.83E+09	1.96E+11	2.72E+09	2.33	12.97
S106	27.56	0.77	13.58	1.75	6.75	0.67	6.71	10.05	5.46	15.66	69.24	1645.76	220.36	113.77	3.39	3.09	2.09E+09	2.54E+11	4.97E+09	2.16	12.95
S109	27.13	0.84	15.13	1.25	6.76	0.59	6.09	10.30	8.03	11.54	54.94	1693.08	205.42	127.84	7.48	2.93	2.89E+09	2.44E+11	1.69E+09	6.44	15.81
S110	26.59	1.01	15.20	0.79	6.80	0.61	6.12	10.01	5.68	4.91	47.54	1081.53	169.12	141.45	14.09	1.80	1.83E+09	2.30E+11	4.55E+09	5.90	16.55
S123	41.89	0.92	16.47	0.72	6.72	0.69	6.88	9.95	11.79	8.78	71.47	1769.63	266.23	238.94	13.27	5.14	6.17E+09	1.44E+11	5.73E+08	3.50	19.24
S124	36.01	0.91	16.06	0.60	6.33	0.67	6.86	10.27	5.55	11.08	56.74	1626.52	226.86	145.74	10.32	3.86	5.75E+09	9.06E+10	1.99E+09	3.30	18.00
S126	36.30	0.81	17.70	0.38	6.86	0.60	5.98	10.04	7.95	7.32	47.77	1365.01	219.47	192.81	9.19	2.41	8.27E+09	7.73E+10	1.84E+09	3.60	14.20
S127	43.53	0.83	18.10	0.74	6.89	0.70	7.32	10.42	8.27	12.92	77.64	2093.15	332.62	173.46	6.98	3.11	3.22E+09	1.00E+10	1.84E+09	4.10	12.72
S128	41.26	0.80	17.05	0.61	6.78	0.65	6.54	10.02	8.20	9.65	57.00	1639.22	259.76	155.71	7.47	3.33	3.27E+09	3.78E+10	2.85E+08	3.40	17.30
S129	40.46	0.83	16.95	0.84	7.03	0.64	6.58	10.20	10.65	12.68	63.53	1611.04	267.44	190.08	5.49	2.52	3.07E+09	1.36E+11	2.10E+09	3.20	13.60
S130	32.91	0.93	15.70	1.26	6.57	0.64	6.57	10.22	6.62	13.26	48.76	1632.90	216.64	133.60	6.67	3.15	7.03E+09	1.51E+11	2.27E+09	4.60	16.40
S133	33.64	1.02	14.61	1.62	6.99	0.60	5.82	9.67	6.91	9.64	53.61	1576.98	237.45	153.48	5.53	3.58	6.09E+09	1.63E+11	2.45E+09	0.70	11.60
S134	35.64	0.91	15.62	0.65	7.09	0.65	6.79	10.45	9.37	11.30	81.32	1833.31	321.50	212.16	9.52	3.20	1.05E+10	1.46E+11	3.64E+09	4.50	15.20
S135	37.85	0.92	14.67	0.72	6.84	0.64	6.29	9.81	10.05	5.04	65.23	1573.33	241.90	193.44	11.31	2.89	4.82E+09	1.70E+11	1.17E+09	2.70	18.52
S136	39.82	0.97	14.87	1.33	6.79	0.64	6.38	9.96	6.33	11.99	59.39	1602.79	238.72	126.40	6.14	2.64	5.67E+09	2.34E+11	1.21E+07	2.50	13.90
S137	35.19	0.95	14.77	1.29	6.83	0.64	6.28	9.85	6.60	8.58	61.47	1676.15	224.98	149.63	9.57	2.94	1.17E+10	1.50E+11	3.33E+09	3.30	22.80
S138	39.97	0.86	15.09	1.99	6.82	0.64	6.16	9.64	8.97	5.01	58.65	1442.43	235.34	170.49	9.90	2.66	5.97E+09	1.07E+11	1.18E+07	3.70	13.70
S139	32.51	0.79	16.08	0.77	6.40	0.67	6.56	9.86	5.72	8.61	50.34	1431.31	181.57	125.60	7.55	1.98	8.49E+09	1.58E+11	3.34E+09	2.40	19.80
S140	29.36	0.83	15.46	0.89	6.67	0.68	6.85	10.10	6.82	5.29	53.81	1232.05	167.23	227.34	17.71	2.14	4.37E+09	1.32E+11	1.82E+09	2.40	12.13
S141	42.96	0.91	16.20	0.24	6.96	0.67	6.84	10.17	12.13	3.70	80.82	1739.37	299.69	203.07	14.26	3.11	6.41E+09	1.75E+11	1.83E+09	1.40	14.90
S142	38.60	0.85	14.10	1.38	6.59	0.62	6.27	10.05	8.81	8.62	51.51	1518.47	214.31	129.57	7.18	3.17	4.89E+09	1.45E+11	1.55E+09	3.30	13.90
S157	36.31	0.90	12.58	1.10	7.12	0.66	6.67	10.09	5.52	11.86	132.00	1755.40	302.07	184.10	5.66	3.54	4.08E+09	2.09E+11	2.10E+09	2.60	16.10
S158	36.15	0.80	13.54	1.10	7.23	0.68	7.01	10.35	9.11	11.63	82.23	1796.92	278.15	198.56	6.42	3.59	1.70E+09	1.40E+11	1.95E+09	1.10	15.40
S159	40.01	0.85	12.23	1.75	6.95	0.71	7.12	9.99	9.89	2.82	94.06	1831.97	328.21	223.16	17.07	3.96	4.03E+09	1.41E+11	1.64E+09	1.40	17.90
S160	36.28	0.93	12.47	1.18	7.13	0.66	6.56	9.95	7.17	10.57	71.05	1636.66	283.51	151.42	4.41	3.55	4.51E+09	2.87E+11	1.40E+09	0.60	11.90
S161	34.51	0.77	15.05	0.96	6.73	0.65	6.33	9.74	8.77	5.91	51.21	1547.23	207.31	145.83	9.49	3.41	4.98E+09	1.93E+11	3.82E+09	2.90	17.00
S162	38.09	0.90	12.68	1.21	6.72	0.61	6.09	10.00	5.81	2.38	54.09	1397.36	211.27	158.27	12.40	2.19	2.22E+09	2.46E+11	2.70E+09	2.20	16.00
S163	33.92	0.95	13.29	0.75	6.87	0.66	6.48	9.85	5.38	9.09	55.13	1682.44	237.88	132.48	5.94	3.69	3.87E+09	2.04E+11	3.99E+09	2.70	14.98
S164	37.93	0.83	11.83	1.19	6.82	0.66	6.53	9.86	10.55	11.12	62.54	1824.83	264.77	144.37	4.15	2.75	9.05E+09	1.87E+11	3.71E+09	2.20	16.50
S165	36.17	0.86	12.32	1.93	6.81	0.71	6.95	9.83	10.46	4.10	68.54	1806.26	304.28	202.86	18.56	3.98	2.18E+09	1.71E+11	2.16E+09	1.90	19.60
S166	37.10	0.92	13.58	1.14	6.96	0.69	6.89	9.95	7.72	11.32	62.34	1807.64	292.30	157.75	7.69	4.40	3.57E+09	3.96E+11	2.85E+09	0.70	12.30
S169	33.97	0.91	15.13	1.06	6.61	0.60	5.78	9.66	6.63	6.09	60.71	1476.59	203.06	115.57	5.11	3.49	3.15E+09	2.49E+11	3.40E+09	1.40	8.22
S170	35.27	0.85	15.20	0.72	6.76	0.60	5.87	9.71	9.62	8.61	49.65	1370.97	204.05	123.07	2.77	2.46	2.10E+09	1.36E+11	1.20E+09	0.60	16.14
S183	44.43	1.08	16.47	0.71	6.19	0.60	6.05	10.03	10.63	8.83	44.64	1516.20	201.70	99.58	0.32	3.21	4.59E+09	2.56E+11	1.68E+09	4.01	8.95
S184	50.82	0.98	16.06	1.30	6.72	0.66	7.20	10.90	14.07	17.93	79.94	2000.09	313.71	143.64	2.15	3.97	2.46E+09	3.90E+11	2.00E+09	3.64	9.31

S186	46.65	0.88	17.70	1.28	6.49	0.59	5.84	9.90	6.44	10.31	45.96	1540.71	217.82	91.85	4.95	2.92	4.70E+09	2.63E+11	4.08E+09	4.73	11.14
S187	48.51	0.84	18.10	1.34	6.80	0.67	6.73	10.07	4.69	23.30	70.64	1722.27	256.44	121.20	-2.05	2.86	4.42E+09	4.10E+11	2.61E+09	6.70	12.59
S188	48.47	1.02	17.05	1.00	6.43	0.69	7.10	10.32	12.44	7.21	59.56	1467.61	208.23	174.63	9.06	2.99	3.07E+09	3.05E+11	2.85E+09	5.21	12.23
S189	51.16	0.92	16.95	1.13	6.54	0.72	7.40	10.22	9.86	16.77	67.59	1969.24	290.92	103.57	-3.06	2.62	5.76E+09	2.79E+11	4.10E+09	4.66	7.00
S190	50.20	0.94	15.70	0.38	6.83	0.74	7.81	10.51	10.44	21.98	77.59	2108.15	319.75	142.03	0.73	4.63	4.98E+09	5.61E+11	6.49E+08	5.75	11.22
S193	44.75	1.02	14.61	0.79	6.82	0.62	6.32	10.26	10.23	16.83	66.64	1709.41	238.67	116.64	-0.63	3.58	2.99E+09	3.32E+11	2.82E+09	6.71	13.70
S194	44.75	1.05	15.62	0.82	6.63	0.62	6.20	10.18	11.70	7.89	79.22	1603.50	268.49	150.13	8.24	4.10	3.99E+09	4.56E+11	1.48E+09	2.74	11.77
S195	51.01	1.04	14.67	0.60	6.66	0.70	7.21	10.37	17.25	17.44	89.34	2147.03	318.58	123.30	-1.75	3.90	5.48E+09	3.51E+11	1.72E+09	4.08	9.97
S196	49.77	1.14	14.87	0.86	6.78	0.70	7.19	10.32	9.55	20.10	87.73	2041.09	308.34	135.23	-1.51	4.04	5.03E+09	4.04E+11	3.26E+09	5.39	11.22
S197	48.23	1.12	14.77	0.50	6.60	0.65	6.41	10.14	12.78	8.25	65.32	1501.40	243.20	150.24	4.15	3.56	3.10E+09	3.84E+11	1.28E+09	5.62	9.47
S198	45.78	1.00	15.09	0.76	6.63	0.66	6.55	9.93	10.30	13.67	73.89	1782.64	259.24	94.72	-1.96	3.30	2.33E+09	4.44E+11	3.00E+09	5.11	8.93
S199	48.17	0.97	16.08	0.73	6.85	0.65	6.71	10.30	8.66	18.60	80.55	2003.91	288.82	106.83	-4.29	4.85	2.52E+09	5.04E+11	2.68E+09	7.97	14.30
S200	48.82	1.08	15.46	0.39	6.88	0.69	7.20	10.63	15.80	7.38	99.24	1897.84	292.64	168.49	8.01	4.83	2.59E+09	4.16E+11	1.61E+09	6.71	12.79
S201	46.32	0.86	16.20	1.52	6.64	0.64	6.68	10.38	12.41	12.22	65.74	1748.00	256.11	86.38	-2.63	3.75	3.48E+09	3.78E+11	1.37E+09	3.02	7.40
S202	43.16	1.04	14.10	0.58	6.61	0.66	6.72	10.21	11.23	13.53	50.58	1670.17	217.47	116.70	0.99	4.12	2.00E+09	3.47E+11	1.67E+09	3.76	10.90
S217	49.27	0.94	12.58	0.68	7.12	0.64	6.53	10.18	11.86	19.30	89.19	1837.16	294.63	150.45	-0.94	3.31	3.67E+09	3.49E+11	2.03E+08	10.55	18.00
S218	44.07	1.08	13.54	0.92	7.14	0.67	6.83	10.16	7.76	6.14	251.29	1677.34	262.07	163.81	10.36	3.67	4.50E+09	2.08E+11	2.45E+08	3.66	10.50
S219	43.31	1.05	12.23	1.47	6.75	0.61	6.15	10.09	10.03	9.36	62.44	1645.01	236.26	83.04	-1.39	2.42	5.91E+09	3.03E+11	1.06E+09	2.94	9.80
S220	44.04	1.01	12.47	1.16	6.89	0.59	6.05	10.34	9.18	12.97	66.56	1717.80	229.02	103.67	-2.02	3.61	3.21E+09	4.22E+11	1.33E+09	3.59	11.92
S221	44.19	1.07	15.05	0.39	6.61	0.61	6.30	10.32	12.11	4.26	72.96	1426.68	192.30	205.07	15.69	4.16	3.86E+09	2.39E+11	2.61E+08	4.56	9.66
S222	42.38	1.03	12.68	0.55	6.61	0.62	6.26	10.07	8.79	9.62	55.04	1568.17	219.24	85.72	-0.58	2.55	2.95E+09	2.08E+11	5.54E+08	4.38	10.49
S223	44.96	0.93	13.29	1.46	6.70	0.64	6.52	10.15	9.59	12.40	63.14	1652.38	213.58	130.61	0.41	4.13	2.40E+09	4.15E+11	1.38E+09	2.98	7.68
S224	46.29	1.03	11.83	1.21	6.73	0.64	6.52	10.15	12.02	6.67	58.74	1616.97	230.89	138.66	5.54	3.10	3.80E+09	3.35E+11	9.06E+08	7.02	15.53
S225	45.92	0.99	12.32	0.51	6.75	0.65	6.57	10.07	12.71	12.27	70.04	1730.07	259.18	111.90	0.32	3.31	6.24E+09	2.31E+11	5.44E+05	4.53	9.33
S226	46.17	1.06	13.58	0.91	6.82	0.63	6.36	10.05	10.18	13.65	58.17	1624.59	228.49	138.03	-1.95	4.42	2.96E+09	2.90E+11	2.50E+09	6.51	12.57
S229	47.06	0.99	15.13	2.14	6.81	0.62	6.42	10.42	17.31	15.13	98.81	1957.84	286.01	169.63	3.09	3.72	3.12E+09	4.23E+11	8.98E+08	4.05	9.11
S230	42.89	1.02	15.20	0.62	6.78	0.59	6.55	11.05	7.58	3.90	62.78	1386.36	184.33	115.50	5.31	2.64	5.05E+09	6.24E+11	3.54E+08	2.29	7.55
S243	29.18	1.03	16.47	0.85	6.97	0.64	6.49	10.13	7.47	8.76	86.17	1647.80	221.67	87.68	3.90	3.49	3.91E+09	3.23E+11	2.17E+09	5.90	12.35
S244	29.27	0.95	16.06	2.12	6.61	0.68	7.29	10.67	8.48	6.07	82.29	1428.15	205.73	86.81	3.05	2.74	2.47E+09	3.73E+11	6.15E+08	4.09	12.68
S245	27.00	1.08	18.23	2.91	6.62	0.64	6.53	10.19	6.02	6.55	77.19	1608.47	211.72	88.31	3.21	2.75	2.02E+09	4.36E+11	2.70E+09	6.14	14.68
S246	26.81	0.94	17.70	1.63	6.86	0.67	6.67	10.01	5.62	6.59	82.08	1798.20	243.73	107.38	8.24	3.12	5.41E+09	3.18E+11	1.64E+09	11.18	20.99
S247	32.91	0.89	18.10	1.17	6.72	0.66	6.64	10.14	5.61	8.05	95.14	1504.48	216.40	83.62	5.61	2.05	6.79E+09	2.99E+11	1.48E+09	5.16	13.15
S248	28.16	1.05	17.05	0.85	6.75	0.64	6.46	10.09	7.05	3.33	87.39	1608.98	210.28	97.72	7.20	3.17	6.52E+09	2.56E+11	3.01E+08	9.33	20.23
S249	24.62	0.92	16.95	0.95	6.54	0.62	6.42	10.37	4.66	6.10	71.47	1612.19	201.81	76.84	1.34	2.58	2.71E+09	2.84E+11	2.25E+09	7.15	14.90
S250	25.91	0.98	15.70	1.37	6.57	0.60	6.17	10.22	2.00	6.46	75.14	1321.67	165.76	75.57	4.77	2.15	4.54E+09	3.49E+11	1.27E+09	4.87	14.14
S253	29.60	0.88	14.61	2.37	6.92	0.58	6.02	10.34	0.55	7.19	80.44	1522.30	208.25	72.58	6.58	2.58	2.40E+09	2.78E+11	1.59E+09	8.05	16.08
S254	27.35	0.80	15.62	1.15	7.13	0.59	5.85	9.91	5.84	7.65	62.17	1470.21	216.02	81.90	1.12	2.68	3.24E+09	3.66E+11	1.10E+09	5.92	11.49
S255	25.90	0.96	14.67	1.04	6.93	0.61	6.31	10.35	5.08	9.96	62.75	1641.23	237.75	96.24	1.31	2.26	1.72E+09	2.74E+11	2.26E+09	4.85	12.64
S256	28.30	0.97	14.87	2.14	7.04	0.63	6.48	10.32	0.20	6.68	83.50	1520.62	226.42	89.07	12.80	1.91	1.84E+09	5.56E+11	2.11E+09	6.20	12.18
S257	24.67	0.95	14.77	1.34	6.67	0.61	6.02	9.79	4.55	6.92	68.27	1582.19	238.92	113.20	4.01	2.02	2.90E+09	2.52E+11	1.61E+09	7.79	16.26
S258	24.82	0.99	15.09	11.15	6.88	0.58	5.82	10.04	4.36	7.12	43.34	1136.55	134.32	123.35	5.49	2.31	1.81E+09	1.73E+12	1.03E+09	4.88	13.75
S259	24.38	1.01	16.08	1.57	6.93	0.61	6.13	10.13	4.58	2.84	52.65	1516.35	194.74	92.68	7.91	2.53	4.37E+09	2.02E+11	5.89E+08	7.19	13.35
S260	23.39	0.95	15.46	0.76	6.83	0.60	5.99	10.00	3.95	2.92	41.12	1260.46	141.72	173.97	10.41	2.15	1.62E+09	2.15E+11	9.14E+08	9.06	14.88
S261	24.01	0.93	16.20	0.77	6.69	0.68	7.19	10.61	3.47	10.45	53.66	1568.36	213.57	82.08	0.31	2.68	2.94E+09	5.96E+11	9.60E+08	3.14	9.82
S262	24.90	0.86	14.10	1.21	6.38	0.63	6.58	10.39	5.89	4.39	53.28	1471.09	194.14	75.32	3.51	2.79	2.77E+09	3.74E+11	1.30E+09	4.57	12.84
S277	28.71	0.85	12.58	2.48	7.01	0.64	6.65	10.35	4.04	5.05	86.67	1573.03	227.84	71.75	5.82	2.15	2.46E+09	2.83E+11	1.67E+09	3.08	9.48
S278	28.48	0.94	13.54	1.80	6.94	0.66	6.86	10.39	5.57	6.02	66.00	1680.05	226.67	112.90	4.97	3.18	2.98E+09	3.87E+11	1.71E+09	4.56	11.82
S279	26.11	0.90	12.23	0.81	6.72	0.65	6.59	10.12	4.15	12.53	75.85	1626.02	231.22	77.65	-0.95	2.58	3.38E+09	3.25E+11	2.52E+09	4.59	13.43
S280	26.36	0.82	12.47	0.90	6.48	0.58	6.02	10.37	4.69	6.49	62.02	1384.07	178.17	62.19	1.23	2.20	3.71E+09	3.47E+11	7.68E+08	3.92	14.47

S281	27.84	1.02	15.05	1.13	6.91	0.63	6.44	10.20	8.19	7.01	67.75	1529.13	212.08	113.18	6.11	2.59	4.26E+09	3.02E+11	1.09E+09	4.91	11.96
S282	24.29	1.03	12.68	1.72	6.57	0.55	5.47	10.02	2.71	7.39	50.24	1217.72	148.02	83.71	2.37	1.56	2.76E+09	2.95E+11	1.78E+09	3.77	10.47
S283	28.00	1.01	13.29	1.20	6.90	0.66	6.95	10.50	6.55	11.53	74.55	1779.46	241.57	116.93	3.82	3.71	5.86E+09	4.21E+11	1.06E+09	5.92	18.21
S284	27.70	1.02	11.83	1.84	6.58	0.63	6.39	10.14	6.18	4.63	65.32	1578.71	193.57	109.77	4.69	2.86	4.49E+09	2.96E+11	7.35E+08	2.94	11.96
S285	27.86	0.89	12.32	0.85	6.91	0.70	7.27	10.35	5.19	11.71	90.92	1815.99	250.00	126.60	6.95	3.20	5.18E+09	3.65E+11	2.12E+08	9.83	18.97
S286	28.17	1.02	13.58	1.62	6.75	0.62	6.28	10.15	6.64	9.09	66.80	1564.28	198.91	89.69	0.20	3.10	5.24E+09	3.83E+11	9.09E+08	8.31	20.88
S289	29.48	0.88	15.13	1.74	6.63	0.62	6.45	10.35	4.97	9.27	59.62	1483.74	190.12	74.80	-0.72	2.24	2.44E+09	3.57E+11	3.04E+09	5.22	12.10
S290	23.98	0.92	15.20	3.80	6.82	0.60	6.23	10.31	5.04	2.71	78.65	1368.17	167.63	96.53	6.05	2.43	4.89E+09	2.82E+11	1.88E+09	3.45	12.65
S303	37.03	0.97	16.47	0.91	6.67	0.60	6.27	10.39	6.41	7.97	75.24	1872.68	239.81	94.61	0.98	3.33	2.70E+09	1.87E+11	3.14E+09	3.65	7.03
S304	40.67	0.84	16.06	1.76	6.52	0.62	6.52	10.50	8.34	11.14	72.98	1848.00	238.15	92.00	-0.14	2.80	2.86E+09	3.34E+11	2.47E+09	7.73	13.65
S306	38.58	0.88	17.70	1.85	6.72	0.64	6.74	10.48	7.08	11.61	76.36	2035.63	254.66	121.55	1.88	4.15	2.43E+09	3.78E+11	1.87E+09	10.35	15.36
S307	42.81	0.73	18.10	1.12	6.52	0.64	6.44	10.01	6.92	14.02	68.98	1677.64	242.76	133.81	4.66	2.13	4.05E+09	4.05E+11	4.69E+09	6.91	9.94
S308	38.76	0.82	17.05	1.26	6.69	0.68	6.99	10.31	5.34	10.30	80.14	1939.97	243.74	93.41	3.88	3.27	2.72E+09	4.94E+11	2.92E+09	6.64	11.75
S309	39.29	0.89	16.95	1.52	6.50	0.63	6.35	10.03	10.56	13.56	69.73	2104.29	255.34	93.56	-1.99	5.12	4.91E+09	3.90E+11	5.06E+09	8.42	14.19
S310	37.22	0.83	15.70	0.68	6.63	0.66	6.71	10.20	9.61	8.93	54.31	2042.22	233.05	107.03	4.42	2.82	2.67E+09	4.16E+11	2.15E+09	6.66	10.87
S313	45.20	0.85	14.61	0.74	6.64	0.65	6.77	10.38	7.43	14.96	91.14	2189.72	326.35	109.18	-0.82	3.43	3.67E+09	3.07E+11	5.60E+09	7.34	13.59
S314	40.17	0.95	15.62	2.82	7.03	0.66	6.58	9.94	6.93	7.79	77.06	1999.65	280.05	100.29	6.53	5.13	3.34E+09	3.40E+11	2.00E+09	6.44	10.69
S315	40.96	0.87	14.67	2.59	6.85	0.66	6.73	10.24	7.93	12.86	70.45	2280.13	331.84	99.52	-1.01	4.07	5.97E+09	2.00E+11	4.04E+09	4.30	8.91
S316	37.69	0.92	14.87	1.85	6.82	0.67	6.90	10.36	5.28	14.89	75.04	2099.74	278.23	104.29	-0.77	2.92	3.24E+09	4.71E+11	2.32E+09	3.29	5.83
S317	40.20	0.83	14.77	3.76	6.56	0.64	6.31	9.90	7.99	10.45	55.24	1797.80	219.86	90.96	1.66	3.73	3.00E+09	2.73E+11	3.56E+09	7.62	12.18
S318	40.39	0.92	15.09	1.88	6.08	0.63	6.14	9.82	5.03	13.88	67.28	1937.34	252.22	105.98	-2.08	3.97	3.97E+09	3.36E+11	8.44E+08	9.03	16.26
S319	41.18	0.77	16.08	1.21	6.96	0.66	6.66	10.14	9.03	11.07	68.05	2193.35	284.19	100.60	-1.01	3.88	3.16E+09	3.26E+11	4.57E+09	8.97	13.50
S320	40.32	0.82	15.46	5.28	6.80	0.66	6.73	10.17	8.54	3.36	66.48	2015.93	242.09	119.61	12.93	3.96	3.11E+09	3.59E+11	2.81E+09	7.93	14.57
S321	39.47	0.86	16.20	1.07	6.86	0.65	6.66	10.26	8.36	13.57	83.66	2209.96	314.32	107.62	-0.39	3.51	6.57E+09	3.67E+11	4.79E+09	7.30	12.75
S322	39.47	0.85	14.10	0.92	6.76	0.67	6.67	9.98	8.85	12.16	61.64	2075.82	265.82	103.38	1.86	3.46	2.27E+09	3.53E+11	3.09E+09	6.66	12.17
S337	38.98	0.90	12.58	2.01	7.00	0.66	6.56	9.91	6.85	12.08	83.09	2344.36	325.24	108.55	-1.13	3.28	2.79E+09	3.93E+11	4.34E+09	9.75	17.09
S338	41.02	0.80	13.54	1.03	6.81	0.64	6.61	10.39	4.94	11.12	83.44	2154.70	295.86	107.68	0.39	4.03	3.09E+09	4.88E+11	3.12E+09	8.75	15.36
S339	38.54	0.88	12.23	1.00	6.75	0.63	6.87	10.92	6.97	7.06	83.87	2130.89	268.74	88.13	2.39	3.83	5.06E+09	4.27E+11	3.62E+09	10.64	17.93
S340	39.00	0.99	12.47	0.87	6.79	0.68	7.01	10.23	8.06	9.20	71.73	2161.65	287.39	88.47	-0.21	3.05	2.85E+09	4.57E+11	2.16E+09	11.32	16.42
S341	45.06	0.81	15.05	3.69	6.78	0.66	6.67	10.09	7.34	11.62	93.06	2110.65	281.62	97.23	0.36	4.19	2.62E+09	2.95E+11	5.29E+09	7.21	11.75
S342	39.68	0.89	12.68	2.32	6.48	0.65	6.48	9.98	7.76	9.65	64.34	1828.41	236.13	78.66	1.19	4.27	4.19E+09	6.36E+11	1.87E+09	8.82	14.30
S343	42.09	0.94	13.29	1.00	6.81	0.61	6.17	10.13	6.47	10.29	69.66	1933.12	253.28	108.84	-0.88	2.88	3.17E+09	4.33E+11	5.28E+09	8.04	13.07
S344	39.68	0.91	11.83	4.52	6.92	0.67	6.79	10.13	8.46	7.76	63.08	1861.83	240.02	95.63	0.04	3.36	3.21E+09	1.45E+12	1.61E+09	11.84	17.36
S345	41.61	0.86	12.32	1.06	6.58	0.64	6.53	10.17	9.66	10.67	80.98	1878.53	269.91	86.00	0.14	3.96	3.24E+09	4.70E+11	4.32E+09	5.43	11.27
S346	40.55	0.79	13.58	1.43	6.84	0.71	7.31	10.30	8.29	15.51	74.41	2013.37	277.48	98.62	-0.78	3.40	3.38E+09	3.94E+11	2.46E+09	7.03	11.16
S349	41.60	0.85	15.13	2.63	7.01	0.65	6.46	9.94	7.84	13.01	70.60	2264.85	320.86	107.57	-0.17	2.42	3.38E+09	4.52E+11	4.22E+09	9.25	15.28
S350	40.58	0.88	15.20	1.32	6.84	0.64	6.70	10.44	6.37	10.55	73.31	1999.10	273.05	100.06	1.00	4.33	3.62E+09	4.26E+11	2.30E+09	6.60	14.32
average	39.36	0.91	14.87	1.49	6.72	0.65	6.55	10.16	8.99	11.34	71.96	1693.97	241.91	142.91	6.49	2.88	7.26E+09	3.11E+11	2.27E+09	4.89	12.51
minimum	23.39	0.54	11.83	0.24	6.08	0.55	5.47	9.64	0.20	2.16	41.12	1073.71	117.31	62.19	-4.29	1.23	1.62E+09	6.77E+09	5.44E+05	0.60	2.70
maximum	63.20	1.18	18.23	11.15	7.23	0.74	7.81	11.07	29.10	67.66	251.29	2344.36	337.32	355.42	32.96	5.14	5.64E+10	1.73E+12	5.60E+09	11.84	27.61
st. deviation	10.93	0.10	1.73	1.09	0.20	0.03	0.41	0.24	4.41	6.82	20.19	247.70	43.58	49.19	6.30	0.89	9.01E+09	1.85E+11	1.23E+09	2.44	4.10
