# Supplementary material for: Functional Traits and Spatio-Temporal Structure of a Major Group of Soil Protists (Rhizaria: Cercozoa) in a Temperate Grassland
Source: Front Microbiol. 2019 Jun 11;10:1332. doi: 10.3389/fmicb.2019.01332 (PMC6579879; doi:10.3389/fmicb.2019.01332)
Supplement: Supplementary file 1 [file Data_Sheet_1.zip › Data Sheet 1/FioreDonnoSupplMat/TableS2Barcodes.pdf]

Table S2. Combinations of barcodes used in this study, with the corresponding samples.

Forward	Reverse	Sample	Forward	Reverse	Sample	Forward	Reverse	Sample
CTACATCT	ACACGACG	S003	ACACAGCA	GAGTGACG	S075	GAGCTAGT	AGATCAGC	S040
CAGCATGA	ACACGACG	S004	CTACATCT	GCATGCGT	S076	GAGACATA	GAGATCAT	S099
CAGATGTC	ACACGACG	S063	CAGCATGA	GCATGCGT	S135	CTGCTGCA	GCGTCGAC	S100
ATGACTCT	ACACGACG	S064	CAGATGTC	GCATGCGT	S136	CTACTAGC	TAGTCGCT	S159
ATAGCAGT	ACACGACG	S123	ATGACTCT	GCATGCGT	S195	CTACTAGC	AGACTGAC	S160
ATAGAGTC	ACACGACG	S124	ATAGCAGT	GCATGCGT	S196	GCAGATGC	GAGTGACG	S219
ACGATACA	ACACGACG	S183	ATAGAGTC	GCATGCGT	S255	GCACTATA	TAGCGCAG	S220
ACACAGCA	ACACGACG	S184	ACGATACA	GCATGCGT	S256	GCACTATA	ACGAGCGC	S279
CTACATCT	ACGAGCGC	S243	ACACAGCA	GCATGCGT	S315	GAGCTAGT	AGCATACT	S280
CAGCATGA	ACGAGCGC	S244	CTACATCT	GCGTCGAC	S316	GAGACATA	GAGTGACG	S339
CAGATGTC	ACGAGCGC	S303	CAGCATGA	GCGTCGAC	S017	CTGCTGCA	TACATGCG	S340
ATGACTCT	ACGAGCGC	S304	CAGATGTC	GCGTCGAC	S018	CTGCTGCA	ACACGACG	S041
ATAGCAGT	ACGAGCGC	S005	ATGACTCT	GCGTCGAC	S077	CTACTAGC	AGATCAGC	S042
ATAGAGTC	ACGAGCGC	S006	ATAGCAGT	GCGTCGAC	S078	GCAGATGC	GCATGCGT	S101
ACGATACA	ACGAGCGC	S065	ATAGAGTC	GCGTCGAC	S137	GCACTATA	TAGTCGCT	S102
ACACAGCA	ACGAGCGC	S066	ACGATACA	GCGTCGAC	S138	GCACTATA	AGACTGAC	S161
CAGCATGA	AGACTGAC	S126	ACACAGCA	GCGTCGAC	S197	GAGCTAGT	AGCTGCAC	S162
ATGACTCT	AGACTGAC	S186	CTACATCT	TACATGCG	S198	GAGACATA	GCATGCGT	S221
ATAGCAGT	AGACTGAC	S245	CAGCATGA	TACATGCG	S257	CTGCTGCA	TAGCGCAG	S222
ATAGAGTC	AGACTGAC	S246	CAGATGTC	TACATGCG	S258	CTGCTGCA	ACGAGCGC	S281
ACACAGCA	AGACTGAC	S306	ATGACTCT	TACATGCG	S317	GCAGATGC	GCGTCGAC	S282
CTACATCT	AGATCAGC	S007	ATAGCAGT	TACATGCG	S318	GCACTATA	TCGCTAGT	S341
CAGCATGA	AGATCAGC	S008	ATAGAGTC	TACATGCG	S019	GCACTATA	AGATCAGC	S342
CAGATGTC	AGATCAGC	S067	ACGATACA	TACATGCG	S020	GAGCTAGT	GAGATCAT	S043
ATGACTCT	AGATCAGC	S068	ACACAGCA	TACATGCG	S079	GAGACATA	GCGTCGAC	S044
ATAGCAGT	AGATCAGC	S127	CTACATCT	TAGCGCAG	S080	CTGCTGCA	TAGTCGCT	S103
ATAGAGTC	AGATCAGC	S128	CAGCATGA	TAGCGCAG	S139	CTGCTGCA	AGACTGAC	S104
ACGATACA	AGATCAGC	S187	CAGATGTC	TAGCGCAG	S140	CTACTAGC	AGCTGCAC	S163
ACACAGCA	AGATCAGC	S188	ATGACTCT	TAGCGCAG	S199	GCAGATGC	TACATGCG	S164
CTACATCT	AGCATACT	S247	ATAGCAGT	TAGCGCAG	S200	GCAGATGC	ACACGACG	S223
CAGCATGA	AGCATACT	S248	ATAGAGTC	TAGCGCAG	S259	GCACTATA	AGCATACT	S224
CAGATGTC	AGCATACT	S307	ACGATACA	TAGCGCAG	S260	GAGCTAGT	GAGTGACG	S283
ATGACTCT	AGCATACT	S308	CTACATCT	TAGTCGCT	S319	GAGACATA	TACATGCG	S284
ATAGCAGT	AGCATACT	S009	CAGCATGA	TAGTCGCT	S320	GAGACATA	ACACGACG	S343
ATAGAGTC	AGCATACT	S010	CAGATGTC	TAGTCGCT	S021	CTGCTGCA	AGATCAGC	S344
ACGATACA	AGCATACT	S069	ATGACTCT	TAGTCGCT	S022	CTACTAGC	GAGATCAT	S045
ACACAGCA	AGCATACT	S070	ATAGCAGT	TAGTCGCT	S081	GCAGATGC	TAGCGCAG	S046
CTACATCT	AGCTGCAC	S129	ATAGAGTC	TAGTCGCT	S082	GCAGATGC	ACGAGCGC	S105
CAGCATGA	AGCTGCAC	S130	ACGATACA	TAGTCGCT	S141	GCACTATA	AGCTGCAC	S106
CAGATGTC	AGCTGCAC	S189	ACACAGCA	TAGTCGCT	S142	GAGCTAGT	GCATGCGT	S165
ATGACTCT	AGCTGCAC	S190	GCAGATGC	AGCATACT	S201	GAGACATA	TAGCGCAG	S166
ATAGCAGT	AGCTGCAC	S249	GCACTATA	GCATGCGT	S202	GAGACATA	ACGAGCGC	S225
ATAGAGTC	AGCTGCAC	S250	GAGCTAGT	TAGCGCAG	S261	CTGCTGCA	AGCATACT	S226
ACGATACA	AGCTGCAC	S309	GAGCTAGT	ACGAGCGC	S262	CTACTAGC	GAGTGACG	S285
CTACATCT	GAGATCAT	S310	GAGACATA	AGCATACT	S321	GCAGATGC	TAGTCGCT	S286
CAGCATGA	GAGATCAT	S013	CTGCTGCA	GAGTGACG	S322	GCAGATGC	AGACTGAC	S345
CAGATGTC	GAGATCAT	S014	CTACTAGC	TACATGCG	S037	GCACTATA	GAGATCAT	S346
ATGACTCT	GAGATCAT	S073	CTACTAGC	ACACGACG	S038	GAGCTAGT	GCGTCGAC	S049
ATAGCAGT	GAGATCAT	S074	GCAGATGC	AGCTGCAC	S097	GAGACATA	TAGTCGCT	S050
ATAGAGTC	GAGATCAT	S133	GCACTATA	GCGTCGAC	S098	GAGACATA	AGACTGAC	S109
ACGATACA	GAGATCAT	S134	GAGCTAGT	TAGTCGCT	S157	CTGCTGCA	AGCTGCAC	S110
ACACAGCA	GAGATCAT	S193	GAGCTAGT	AGACTGAC	S158	CTACTAGC	GCATGCGT	S169
CTACATCT	GAGTGACG	S194	GAGACATA	AGCTGCAC	S217	GCAGATGC	TCGCTAGT	S170
CAGCATGA	GAGTGACG	S253	CTGCTGCA	GCATGCGT	S218	GCAGATGC	AGATCAGC	S229
CAGATGTC	GAGTGACG	S254	CTACTAGC	TAGCGCAG	S277	CTACATCT	GAGTGACG	S230
ATGACTCT	GAGTGACG	S313	CTACTAGC	ACGAGCGC	S278	GAGCTAGT	TACATGCG	S289
ATAGCAGT	GAGTGACG	S314	GCAGATGC	GAGATCAT	S337	GAGCTAGT	ACACGACG	S290
ATAGAGTC	GAGTGACG	S015	GCACTATA	TACATGCG	S338	GAGACATA	AGATCAGC	S349
ACGATACA	GAGTGACG	S016	GCACTATA	ACACGACG	S039	CTGCTGCA	GAGATCAT	S350
CTGCTGCA	TCGCTAGT	Mock Cerco						
