# Supplementary material for: Functional Traits and Spatio-Temporal Structure of a Major Group of Soil Protists (Rhizaria: Cercozoa) in a Temperate Grassland
Source: Front Microbiol. 2019 Jun 11;10:1332. doi: 10.3389/fmicb.2019.01332 (PMC6579879; doi:10.3389/fmicb.2019.01332)
Supplement: Supplementary file 1 [file Data_Sheet_1.zip › Data Sheet 1/FioreDonnoSupplMat/TableS4RefFunctionalGroups.pdf]

Table S4. References for the functional traits of the cercozoan taxa identified in this study.

SUB-PHYLUM	CLASS	ORDER	FAMILY	GENUS	NUTRITION	MORPHOLOGY	LOCOMOTION	REFERENCES
Endomyxa					N/A	N/A	N/A	
	Phytomyxea[1]				N/A	N/A	N/A	
		Plasmodiophorida			N/A	N/A	N/A	
			Incertae sedis	*Woronina*	parasite (not plant)	flagellate (intracellular)	non motile endoparasite	Neuhauser et al. 2014
			Polymyxa -lineage	*Ligniera, Polymyxa*	plant parasite	flagellate (intracellular)	non motile endoparasite	Neuhauser et al. 2014
			Spongospora -lineage	*Spongospora*	plant parasite	flagellate (intracellular)	non motile endoparasite	Neuhauser et al. 2014
Filosa					N/A	N/A	N/A	
	Novel clade 10				N/A	N/A	N/A	
		Tremulida			N/A	N/A	N/A	
			Tremulidae	*Tremula*	N/A	naked flagellate	creeping/gliding on substrate	Howe et al. 2011
	Novel clade 12				N/A	N/A	N/A	Bass et al. 2009
	Incertae sedis			*Kraken*	bacterivore	naked amoeba	creeping/gliding on substrate	Dumack et al. 2016
	Granofilosea				N/A	N/A	N/A	
			Novel_Clade_Gran-1-2-3-4-5-6		N/A	N/A	N/A	Bass et al. 2009
			Massisteriidae	*Massisteria*	bacterivore	naked amoeboflagellate	creeping/gliding on substrate[1]	Mylnikov et al. 2015
		Cryptofilida	Mesofilidae	*Mesofila*	bacterivore	naked amoeboflagellate	creeping/gliding on substrate[1]	Bass et al. 2009
		Limnofilida	Limnofilidae	*Limnofila*	bacterivore	naked amoeboflagellate	creeping/gliding on substrate	Howe et al. 2011
	Imbricatea				N/A	N/A	N/A	
		Novel_Clade_3			N/A	N/A	N/A	
		Euglyphida			N/A	testate amoeboflagellate/ amoeba (silica)[2]	N/A	Meisterfeld 2000
			Assulinidae	*Assulina*	omnivore	testate amoeboflagellate/ amoeba (silica)	creeping/gliding on substrate	Meisterfeld 2000
			Euglyphidae		omnivore[3]	testate amoeboflagellate/ amoeba (silica)	creeping/gliding on substrate	Meisterfeld 2000
				Euglypha	omnivore	testate amoeboflagellate/ amoeba (silica)	creeping/gliding on substrate	Meisterfeld 2000
			Paulinellidae	*Paulinella*	N/A	testate amoeboflagellate/ amoeba (silica)	creeping/gliding on substrate	Meisterfeld 2000
			Sphenoderiidae	*Trachelocorythion, Sphenoderia*	omnivore	testate amoeboflagellate/ amoeba (silica)	creeping/gliding on substrate	Meisterfeld 2000
			Trinematidae	*Corythion*	omnivore	testate amoeboflagellate/ amoeba (silica)	creeping/gliding on substrate	Meisterfeld 2000
		Incertae sedis	Imbricatea	*Discomonas*	N/A	naked flagellate	creeping/gliding on substrate	Chantangsi et al. 2010
			Nudifilidae		N/A	naked amoeboflagellate	N/A	Howe et al. 2011
				Nudifila	bacterivore	naked amoeboflagellate	creeping/gliding on substrate	Howe et al. 2011
		Marimonadida			N/A	naked amoeboflagellate	N/A (gliding or swimming)	Howe et al. 2011
		Spongomonadida			N/A	N/A	N/A	
			Spongomonadidae		bacterivore	N/A	N/A	Howe et al. 2011; Hibberd 1976; Strüder-Kypke et al. 1998
				Spongomonas	bacterivore	naked flagellate	creeping/gliding on substrate[4]	Howe et al. 2011; Hibberd 1976; Strüder-Kypke et al. 1998
		Thaumatomonadida			N/A	N/A	N/A	
			Peregriniidae	*Peregrinia*	bacterivore	naked amoeboflagellate	creeping/gliding on substrate	Howe et al. 2011
			Thaumatomonadidae		N/A	N/A	N/A	

		Penardeugenia	omnivore	testate amoeboflagellate/ amoeba (silica)	creeping/gliding on substrate	Dumack et al. 2018
		Thaumatomonas, Thaumatomastix	bacterivore	testate amoeboflagellate/ amoeba (silica)	creeping/gliding on substrate	Howe et al. 2011
		Reckertia	bacterivore	testate amoeboflagellate/ amoeba (silica)	creeping/gliding on substrate	Howe et al. 2011
		Esquamula	bacterivore	naked amoeboflagellate	creeping/gliding on substrate	Shiratori et al. 2012
Sarcomonadea			N/A	N/A	N/A	
Cercomonadida			N/A	N/A	N/A	Bass et al. 2009[5]
	Cercomonadidae		N/A	N/A	N/A	Bass et al. 2009[5]
		Cavernomonas	bacterivore	naked flagellate	creeping/gliding on substrate	Bass et al. 2009
		Cercomonas, Eocercomonas, Cholamonas	omnivore	naked amoeboflagellate	creeping/gliding on substrate	Ekelund 1998; Flavin et al. 2000; Geisen et al. 2016
	Paracercomonadidae		bacterivore	naked amoeboflagellate	N/A	
		Metabolomonas	bacterivore	naked amoeboflagellate	creeping/gliding on substrate	Brabender et al. 2012
		Nucleocercomonas	bacterivore	naked amoeboflagellate	freely swimming	Brabender et al. 2012
		Paracercomonas	bacterivore	naked amoeboflagellate	creeping/gliding on substrate	Bass et al. 2009
Glissomonadida			N/A	N/A	N/A	
	Allapsidae	Group-Te, *Allantion, Allapsa, Teretomonas*	bacterivore	naked flagellate	creeping/gliding on substrate	Howe et al. 2009
	Viridiraptoridae	*Viridiraptor*	eukaryvore	naked amoeboflagellate	creeping/gliding on substrate[1]	Hess et al. 2013
	Bodomorphidae	*Bodomorpha*	bacterivore	naked flagellate	creeping/gliding on substrate	Howe et al. 2009
	Clade-T		N/A	N/A	N/A	Howe et al. 2009
	Clade-Y		N/A	N/A	N/A	Howe et al. 2009
	Dujardinidae	*Dujardina*	bacterivore	naked flagellate	creeping/gliding on substrate	Howe et al. 2009
	Proleptomonadidae	*Proleptomonas*	bacterivore[6]	naked flagellate	freely swimming	Vickerman et al. 2002
	Sandonidae	*Clade-N/F-A, Flectomonas, Mollimonas, Neoheteromita, Sandona*	bacterivore	naked flagellate	creeping/gliding on substrate	Howe et al. 2009
Pansomonadida			N/A	naked amoeboflagellate	creeping/gliding on substrate[1]	Howe et al. 2011
	Agitatidae		N/A	naked amoeboflagellate	creeping/gliding on substrate[1]	Howe et al. 2011
		Aurigamonas	omnivore	naked amoeboflagellate	creeping/gliding on substrate[1]	Vieckerman et al. 2005
		Agitata	bacterivore	naked amoeboflagellate	creeping/gliding on substrate[1]	Howe et al. 2011
Thecofilosea			N/A	N/A	N/A	
Cryomonadida			N/A	testate amoeboflagellate/ amoeba/flagellate (organic/agglutinated)	N/A	Cavalier-Smith 1993
	Cryomonas -lineage	*Cryothecomonas*	eukaryvore	testate amoeboflagellate/ amoeba/flagellate (organic/agglutinated)	N/A	Thomsen et al. 1991
	Protaspa -lineage	*Protaspa*	eukaryvore	testate amoeboflagellate/ amoeba/flagellate (organic/agglutinated)	creeping/gliding on substrate	Howe et al. 2011

	Rhogostomidae		omnivore	testate amoeboflagellate/ amoeba/flagellate (organic/agglutinated)	creeping/gliding on substrate	Dumack et al. 2017
		Capsellina	omnivore	testate amoeboflagellate/ amoeba/flagellate (organic/agglutinated)	creeping/gliding on substrate	Howe et al. 2011
		Rhogostoma	omnivore	testate amoeboflagellate/ amoeba/flagellate (organic/agglutinated)	creeping/gliding on substrate	Dumack et al. 2017
Ventricleftida			N/A	testate amoeboflagellate/ amoeba/flagellate (organic/agglutinated)	creeping/gliding on substrate	Howe et al. 2011
	Ventrifissuridae		eukaryvore	testate amoeboflagellate/ amoeba/flagellate (organic/agglutinated)	creeping/gliding on substrate	Howe et al. 2011
		Verrucomonas, Ventrifissura	eukaryvore	testate amoeboflagellate/ amoeba/flagellate (organic/agglutinated)	creeping/gliding on substrate	Howe et al. 2011
Ebriida	Ebridea		eukaryvore	testate amoeboflagellate/ amoeba (silica)	freely swimming	Howe et al. 2011
		TAGIRI-1-2	N/A	N/A	N/A	Hoppenrath et al. 2006
	Ebriacea		eukaryvore	testate amoeboflagellate/ amoeba (silica)	freely swimming	Howe et al. 2011
		Ebria	eukaryvore	naked flagellate	freely swimming	Hoppenrath et al. 2006; Howe et al. 2011
Tectofilosida			N/A	N/A	N/A	
	Pseudodifflugiidae		eukaryvore	testate amoeboflagellate/ amoeba/flagellate (organic/agglutinated)	creeping/gliding on substrate	Howe et al. 2011

[1] Also possessing an ephemeral flagellate stage (Phytomyxea) or an amoeboid feeding form and a flagellate locomotive form (some Granofilosea,Viridiraptoridae)

[2] Eulgyphida: There are only two rare exceptions to the silica-scales bearing, *Ovulinata* and *Micropyxidiella,* here neglected.

[3] Euglyphidae nutrition: "herbivore" (=our eukaryvore) in Meisterfeld (2000), algivore (Seppey et al. 2017), *Trinema, Euglypha, Assulina* and *Trachelocorythion* have been fed with bacteria only (Wyzelich et al. 2002), so we consider the family as omnivore.

[4] *Spongomonas* is included in the creeping/gliding category, although it is attached to the substrate.

[5] Cercomonadida are described as bacterivore (Bass et al. 2009) but evidence is accumulating towards myco- and algophagy (references given).

[6] *Proleptomonas* was described as osmotroph (Vickerman et al. 2002), but the ATCC strains 50735 and is fed with bacteria.

Bass D, Howe AT, Mylnikov AP, Vickerman K, Chao EE, Smallbone JE, Snell J, Cabral CJ & Cavalier-Smith T. 2009. Phylogeny and classification of Cercomonadida (Protozoa, Cercozoa): *Cercomonas* , *Eocercomonas* , *Paracercomonas* , and *Cavernomonas* gen. nov. *Protist* , 160:483-521. doi:10.1016/j.protis.2009.01.004.

Brabender M, Keve Kiss Á, Domonell A, Nitsche F & Arndt H. 2012. Phylogenetic and morphological diversity of novel soil cercomonad species with a description of two new genera (*Nucleocercomonas* and *Metabolomonas*). *Protist* , 163:495-528. doi:10.1016/j.protis.2012.02.002.

Cavalier-Smith T. 1993. Kingdom *Protozoa* and its 18 phyla. *Microbiol Rev* , 57:953-994.

Chantangsi C & Leander BS. 2010. An SSU rDNA barcoding approach to the diversity of marine interstitial cercozoans, including descriptions of four novel genera and nine novel species. *Int J Syst Evol Microbiol* , 60:1962-1977. doi:10.1099/ijs.0.013888-0.

Dumack K, Öztoprak H, Rüger L & Bonkowski M. 2017. Shedding light on the polyphyletic thecate amoeba genus *Plagiophrys* : transition of some of its species to *Rhizaspis* (Tectofilosida, Thecofilosea, Cercozoa) and the establishment of *Sacciforma* gen. nov. and Rhogostomidae fam. nov. (Cryomonadida, Thecofilosea, Cercozoa). *Protist* , 168:92-108. doi:10.1016/j.protis.2016.11.004.

Dumack K, Schuster J, Bass D & Bonkowski M. 2016. A novel lineage of "naked filose amoebae"; *Kraken carinae* gen. nov. sp. nov. (Cercozoa) with a remarkable locomotion by disassembly of its cell body. *Protist* , 167:268-278. doi:10.1016/j.protis.2016.04.002.

Dumack K, Siemensma FJ & Bonkowski M. 2018. Rediscovery of the testate amoeba genus *Penardeugenia* (Thaumatomonadida, Imbricatea). *Protist* , in press

Ekelund F. 1998. Enumeration and abundance of mycophagous protozoa in soil, with special emphasis on heterotrophic flagellates. *Soil Biol Biochem* , 30:1343-1347. doi:10.1016/S0038-0717(97)00266-6.

Flavin M, O'Kelly C, Nerad TA & Wilkinson G. (2000) Cholamonas cyrtodiopsidis gen. n., sp. n. (Cercomonadida), and endo-commensal, mycophagous heterotrophic flagellate with a double kinetid. *Acta Protozoologica* 39:51-60.

Geisen S, Koller R, Hünninghaus M, Dumack K, Urich T & Bonkowski M. 2016. The soil food web revisited: Diverse and widespread mycophagous soil protists. *Soil Biol Biochem* , 94:10-18. doi:10.1016/j.soilbio.2015.11.010.

Hess S & Melkonian M. 2013. The mystery of clade X: *Orciraptor* gen. nov. and *Viridiraptor* gen. nov. are highly specalised, algivorous amoeboflagellates (Glissomonadida, Cercozoa). *Protist* , 164:706747.

Hibberd DJ. 1976. The fine structure of the colonial colorless flagellates *Rhipidodendron splendidum* Stein and *Spongomonas uvella* Stein with special reference to the flagellar apparatus. *J Protozool* , 23:374-385.

Hoppenrath M & Leander BS. 2006. Ebriid Phylogeny and the expansion of the Cercozoa. *Protist* , 157:279-290. doi:10.1016/j.protis.2006.03.002.

Howe AT, Bass D, Scoble JM, Lewis R, Vickerman K, Arndt H & Cavalier-Smith T. 2011. Novel cultured protists identify deep-branching environmental DNA clades of Cercozoa: New genera *Tremula, Micrometopion, Minimassisteria, Nudifila, Peregrinia* . *Protist* , 162:332-372.

Howe AT, Bass D, Vickerman K, Chao EE & Cavalier-Smith T. 2009. Phylogeny, taxonomy, and astounding genetic diversity of Glissomonadida ord. nov., the dominant gliding zooflagellates in soil (Protozoa: Cercozoa). *Protist* , 160:159-189. doi:10.1016/j.protis.2008.11.007.

Meisterfeld R. 2000. Testate amoebae with filopodia. In: *An illustrated guide to the protozoa* (ed. Lee JJ,Leedale G & Bradbury P), Lawrence, Kansas, U.S.A. pp. 1054-1084.

Mylnikov AP, Weber F, Jürgens K & Wylezich C. 2015. *Massisteria marina* has a sister: *Massisteria voersi* sp. nov., a rare species isolated from coastal waters of the Baltic Sea. Eur J Protistol, 51:299-310. doi:10.1016/j.ejop.2015.05.002.

Neuhauser S, Kirchmair M, Bulman SR & Bass D. 2014. Cross-kingdom host shifts of phytomyxid parasites. *BMC Evol Biol* , 14:33. doi:10.1186/1471-2148-14-33.

Seppey CVW, Singer D, Dumack K, Fournier B, Belbahri L, Mitchell EAD & Lara E. 2017. Distribution patterns of soil microbial eukaryotes suggests widespread algivory by phagotrophic protists as an alternative pathway for nutrient cycling. *Soil Biol Biochem* , 112:68-76. doi:10.1016/j.soilbio.2017.05.002.

Shiratori T, Yabuki A & Ishida K-I. 2012. *Esquamula lacrimiformis* , n.g., n. sp., a new member of Thaumatomonads that lacks siliceous scales. *J Euk Microbiol* , 59:527-536. doi:10.1111/j.1550-7408.2012.00635.x.

Strüder-Kypke MC & Hausmann K. 1998. Ultrastructure of the heterotrophic flagellates *Cyathobodo* sp., *Rhipidodendron huxleyi* Kent, 1880, S*pongomonas sacculus* Kent, 1880, and *Spongomonas* sp. *Eur J Protistol* , 34:376-390. doi:10.1016/S0932-4739(98)80007-2.

Thomsen HA, Buck KR, Bolt PA & Garrison DL. 1991. Fine structure and biology of Cryothecomonas gen. nov. (Protista incertae sedis) from the ice biota. *Can J Zool* , 69:1048-1070.

Vickerman K, Le Ray D, Hoef-Emden K & De Jonckheere J. 2002. The soil flagellate *Proleptomonas faecicola* : Cell organisation and phylogeny suggest that the only described free-living trypanosomatid is not a kinetoplastid but has cercomonad affinities. *Protist* , 153:9-24. doi:10.1078/1434-4610-00079.

Vickerman K, Appleton PL, Clarke KJ & Moreira D. 2005. *Aurigamonas solis* n. gen., n. sp., a soil-dwelling predator with unusual helioflagellate organisation and belonging to a novel clade within the Cercozoa. *Protist,* 156:335-354. doi:10.1016/j.protis.2005.07.003.

Wylezich C, Meisterfeld R, Meisterfeld S & Schlegel M. 2002. Phylogenetic analyses of small subunit ribosomal RNA coding regions reveal a monophyletic lineage of euglyphid testate amoebae (order Euglyphida). *J Euk Microbiol* , 49:108.118. doi:10.1111/j.1550-7408.2002.tb00352.x.
