# Supplementary material for: Functional Traits and Spatio-Temporal Structure of a Major Group of Soil Protists (Rhizaria: Cercozoa) in a Temperate Grassland
Source: Front Microbiol. 2019 Jun 11;10:1332. doi: 10.3389/fmicb.2019.01332 (PMC6579879; doi:10.3389/fmicb.2019.01332)
Supplement: Supplementary file 1 [file Data_Sheet_1.zip › Data Sheet 1/FioreDonnoSupplMat/TableS5BetaDiv.pdf]

Table S5. Beta diversity indices calculated for each sampling date.

	April	May	June	August	October	November
**Total dissimilarity (Bray-Curtis, rarefied data)**	0.81	0.81	0.8	0.8	0.8	0.81
**Total dissimilarity (Bray-Curtis, relative data)**	0.81	0.81	0.79	0.79	0.79	0.8
**Total dissimilarity (Sorensen, presence-absence)**	0.53	0.52	0.52	0.45	0.46	0.54
**Turnover (Sorensen, presence-absence)**	0.36	0.38	0.33	0.34	0.34	0.43
**Nestedness (Sorensen, presence-absence)**	0.17	0.15	0.19	0.1	0.13	0.12
