# Supplementary material for: Functional Traits and Spatio-Temporal Structure of a Major Group of Soil Protists (Rhizaria: Cercozoa) in a Temperate Grassland
Source: Front Microbiol. 2019 Jun 11;10:1332. doi: 10.3389/fmicb.2019.01332 (PMC6579879; doi:10.3389/fmicb.2019.01332)
Supplement: Supplementary file 1 [file Data_Sheet_1.zip › Data Sheet 1/FioreDonnoSupplMat/TableS6GLM.pdf]

Table S6. Linear mixed models showing the effects of the environmental predictors on the most abundant 12 cercozoan/endomyxan families (green=bacterivore; blue=plant parasite, brown=omnivore, black=unknown), the morphotype, nutrition and locomotion modes. We give: a) the spatial correlation structure best correcting the starting model according to the AIC; b) the number of models within two AICc units (after model dredging); c) the number of predictors included in all models extracted in a); d) the remaining, highly significant predictors after fitting a model with just the consensus predictors in b), and their effect type (positive or negative); e) their significance level (p values: *<0.05, **<0.01, ***<0.001); f) the R^2 of the fixed effects of the final model with only the highly significant predictors (d); g) the R^2 of the fixed and random effects of the final model with only the highly significant predictors.

Family	Correlation Structure	#Models within 2 AICc units	#Consen-sus predictors	Highly significant (effect type +/-)	Signi-ficance level	R^2 (fixed)	R^2 (total)
**Sandonidae**	NA	2	4	Clay (-)<br>pH (+)<br>Soil Moisture (+)	***<br>***<br>***	0.271	0.282
**Paracercomonadidae**	NA	6	5	Archaeal 16S (-)<br>Clay (+)<br>Soil Moisture (+)	***<br>**<br>***	0.354	0.355
**Cercomonadidae**	NA	13	4	Clay (+)<br>Soil Moisture (+)<br>Extr. Org. Carbon (-)	**<br>***<br>**	0.247	0.299
**Spongomonadidae**	NA	22	2	Extr. Org. Carbon (+)<br>Root Biomass (-)	**<br>**	0.105	0.293
**Unclassified Euglyphida**	NA	3	4	Clay (+)<br>Soil Moisture (-)	***<br>***	0.244	0.244
**Euglyphidae**	NA	2	5	C/N Ratio (+)<br>pH (-)<br>Soil Moisture (-)<br>Soil Organic Carbon (-)<br>Total Nitrogen (-)	***<br>**<br>***<br>***<br>***	0.235	0.265
***Spongospora nasturtii* lineage**	NA	4	4	Microbial Carbon (+)<br>Microbial Nitrogen (-)<br>pH (+)	**<br>**<br>**	0.054	0.054
**Trinematidae**	NA	2	2	Soil Moisture (-)	***	0.263	0.42
**Rhogostomidae**	Rational	17	1	Soil Moisture (-)	***	0.408	0.644
**Allapsidae**	Spherical	3	3	Clay (+)	**	0.042	0.042
***Polymyxa* lineage**	Spherical	16	2	Bacterial cell counts (-)	**	0.017	0.017
**Thaumatomonadidae**	Spherical	6	6	Soil Moisture (+)	***	0.197	0.226
**Morphotype**							
**Naked flagellate**	NA	4	3	Clay (-)<br>pH (+)<br>Soil moisture (+)	***<br>**<br>**	0.189	0.226
**Naked amoeboflagellate**	NA	4	3	Clay (+)<br>Soil moisture (+)	***<br>***	0.315	0.438
**Flagellate / intracellular parasite**	NA	5	2	Bacterial cell counts (-)<br>Total N (+)	**<br>**	0.094	0.094
**Naked amoeba**	NA	23	2	N microbial biomass (-)	**	0.03	0.094
**Testate amoeba/ amoeboflagellate/flagellate (test organic, agglutinated or siliceous)**	NA	21	2	Soil moisture (-)	***	0.381	0.621
**Nutrition mode**							
**Bacterivore[1]**	Exponential	NA	NA	Soil moisture (+)	***	0.29	0.371

**Omnivore**	NA	11	2	Soil moisture (-)	***	0.23	0.344
**Plant parasite**	NA	2	2	Bacterial cell counts (-)<br>C microbial biomass (+)	**<br>**	0.088	0.088
**Parasite (not plant)**	Gaussian	4	3	Total N (+)	**	0.027	0.036
**Eukaryvore**	NA	19	2	Bacterial cell counts (+)	***	0.119	0.187
**Locomotion mode**							
**Creeping/gliding on substrate**	NA	3	3	Bacterial cell counts (+)<br>Total_N (-)	**<br>***	0.091	0.094
**Non-motile endoparasite**	NA	5	2	Bacterial cell counts (-)<br>Total_N (+)	**<br>**	0.094	0.094
**Freely swimming**	NA	1	2	Bulk density (-)	**	0.035	0.035
