# Supplementary material for: Functional Traits and Spatio-Temporal Structure of a Major Group of Soil Protists (Rhizaria: Cercozoa) in a Temperate Grassland
Source: Front Microbiol. 2019 Jun 11;10:1332. doi: 10.3389/fmicb.2019.01332 (PMC6579879; doi:10.3389/fmicb.2019.01332)
Supplement: Supplementary file 1 [file FigS5RAxML_bipartitions.ScalemicCerco869_1447Tree.pdf › Data Sheet 1/FioreDonnoSupplMat/FigS5RAxML_bipartitions.ScalemicCerco869_1447Tree.pdf]

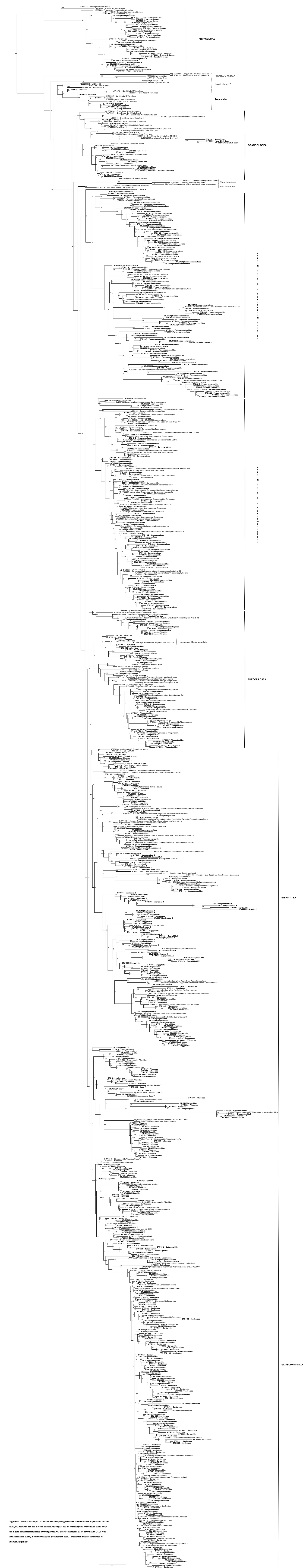


**Figure S5**. Cercozoa/Endomyxa Maximum Likelihood phylogenetic tree, inferred from an alignment of 870 taxa and 1,447 positions. The tree is rooted between Phytomyxea and the remaining taxa. OTUs found in this study are in bold. Main clades are named according to the PR2 database taxonomy, clades for which no OTUs were found are named in gray. Bootstrap values are given for each node. The scale bar indicates the fraction of substitutions per site.
